# A quantitative comparison of cytosolic delivery via different protein uptake systems

**DOI:** 10.1038/s41598-017-13469-y

**Published:** 2017-10-16

**Authors:** Wouter P. R. Verdurmen, Marigona Mazlami, Andreas Plückthun

**Affiliations:** 1 Department of Biochemistry, University of Zurich, Winterthurerstr. 190, 8057 Zurich, Switzerland; 2 Present Address: Department of Biochemistry, Radboud Institute for Molecular Life Sciences (RIMLS), Radboud university medical center, Geert Grooteplein 28, 6525 GA Nijmegen, The Netherlands

## Abstract

Over many years, a variety of delivery systems have been investigated that have the capacity to shuttle macromolecular cargoes, especially proteins, into the cytosol. Due to the lack of an objective way to quantify cytosolic delivery, relative delivery efficiencies of the various transport systems have remained unclear. Here, we demonstrate the use of the biotin ligase assay for a quantitative comparison of protein transport to the cytosol via cell-penetrating peptides, supercharged proteins and bacterial toxins in four different cell lines. The data illustrate large differences in both the total cellular internalization, which denotes any intracellular location including endosomes, and in the cytosolic uptake of the transport systems, with little correlation between the two. Also, we found significant differences between the cell lines. In general, protein transport systems based on cell-penetrating peptides show a modest total uptake, and mostly do not deliver cargo to the cytosol. Systems based on bacterial toxins show a modest receptor-mediated internalization but an efficient delivery to the cytosol. Supercharged proteins, on the contrary, are not receptor-specific and lead to massive total internalization into endosomes, but only low amounts end up in the cytosol.

## Introduction

In general, cells do not permit access of polar macromolecules such as proteins or nucleic acids to their cytosol, and phospholipid membranes constitute an effective barrier. Nonetheless, over the years a number of systems have been reported to give cytoplasmic access to biomacromolecules, most notably cell-penetrating peptides^1,2^, supercharged proteins^3,4^, and bacterial toxins^5,6^. Many other, often non-protein based systems, including many different types of nanoparticles, have been reported to be capable of this as well^7–9^. A confounding feature especially of mammalian cells is their ability of endocytosis, which makes a clear distinction between endosomal and cytosolic localization of the taken-up molecules very critical.

**Figure 1:**
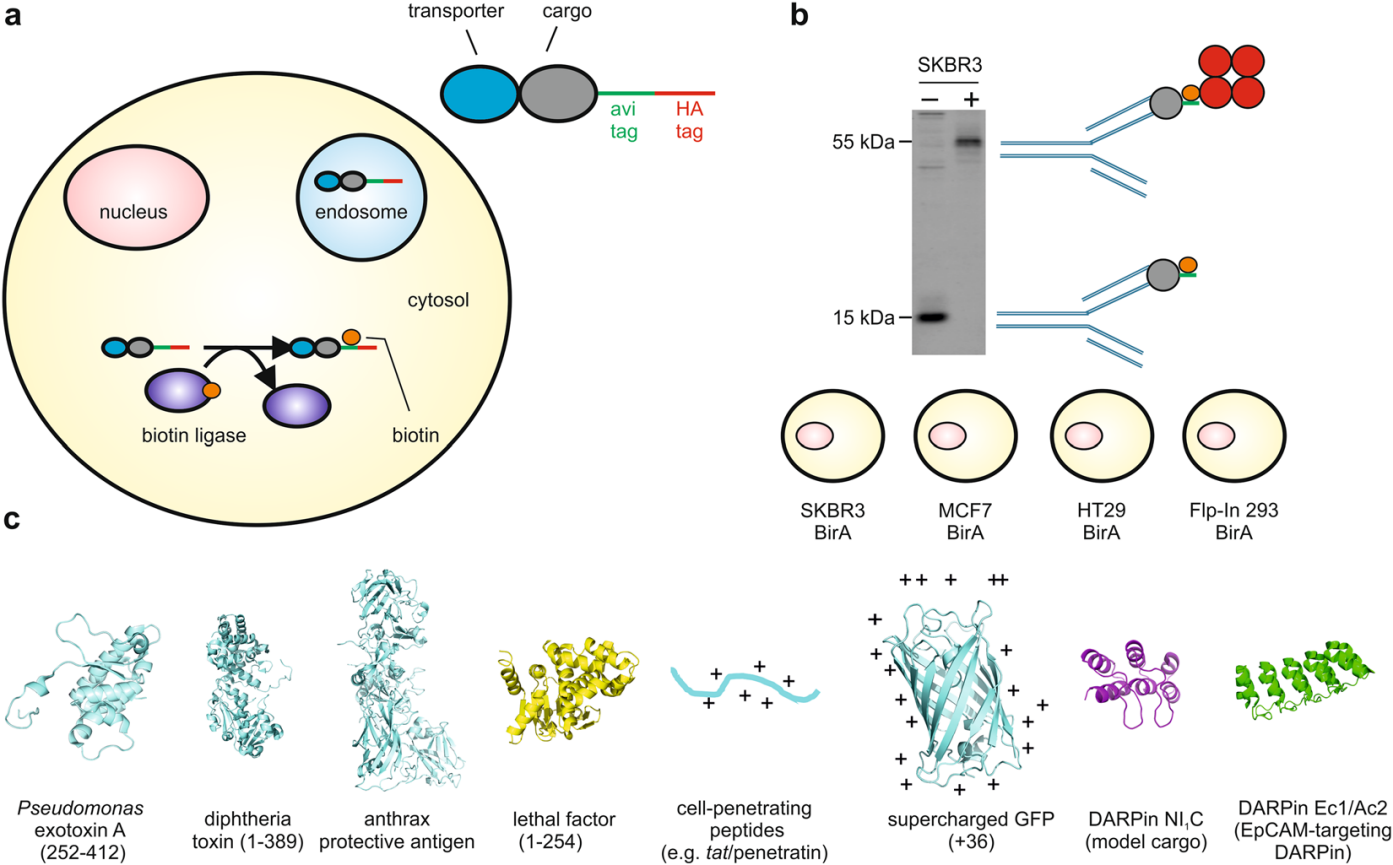
The biotin ligase assay and protein uptake system components. (**a**) Schematic representation of the biotin ligase assay. (**b**) A non-selected DARPin with an avi tag was expressed in the cytosol of SKBR3 cells stably overexpressing prokaryotic biotin ligase. The lysate was either pre-incubated with streptavidin (+) or not (−) before being probed using anti-DARPin serum by western blotting. The biotinylated protein is symbolized by a grey circle, biotin by an orange circle, and tetrameric streptavidin by red circles. The blot image has been cropped for conciseness. The full-size blot is presented in Supplementary Fig. 5. The four cell lines, which were modified to stably overexpress biotin ligase, are listed. (**c**) Schematic structural models of the components of the transporter-cargo fusion proteins that were assessed for cytosolic delivery in this study. Not drawn to scale. From left to right, crystal structures used were PDB ID: 1IKQ for *Pseudomonas* exotoxin A^51^, PDB ID: 1MDT for diphtheria toxin^52^, PDB ID: 1ACC for anthrax protective antigen^53^, PDB ID: 1J7N for lethal factor^54^, PDB ID: 1EMA for green fluorescent protein^55^ and PDB ID: 2XEE for the designed ankyrin repeat proteins^56^. The structure of the model cargo NI_1_C is a model generated by removing two of the three internal repeats from the structure of the full consensus DARPin with three internal repeats (PDB ID: 2XEE). DARPin, designed ankyrin repeat protein. EpCAM, epithelial cell adhesion molecule. Diphtheria toxin (1-389) contains the mutation G79D inactivating the catalytic activity.

However, up to this point, an objective comparison of the efficiency of cytosolic delivery of different transporters is lacking. The main reason is that a quantitative comparison of the fraction of delivered cargo that reaches the cytosol, as opposed to being sequestered in endosomes, has been difficult to achieve. Recently, we showed that it is possible to use a biotin ligase (BirA) assay for the objective quantification of cytosolic delivery^6^. The principle of the assay is that a 15-amino-acid tag, the avi tag, will become biotinylated when it encounters the prokaryotic BirA, which is exclusively overexpressed in the cytosol of the reporter cell lines^6^ (Fig. 1a). Importantly, the avi tag is not a substrate of endogenous eukaryotic biotin ligases. Biotinylation of an overexpressed avi-tagged protein was always 100% in the presence of a very high continuous production, with never any unbiotinylated protein detectable in the steady state, demonstrating the high rate and high efficiency of the avi tag biotinylation in the presence of biotin ligase. A harsh lysis method ensures that no biotinylation activity occurs after lysis^10^. The assay is highly sensitive because of the extremely high affinity between biotin and streptavidin, which is used for detection in western blots, and which is furthermore a sensitive method in general. The assay can be used for the detection of amounts that reflect cytosolic concentrations from low nanomolar to high micromolar. The quantitation gives a linear response over a wide range^6^. In some cases the lower detection limit can be compromised due to background signals from proteins that non-specifically interact with streptavidin, in which case an anti-biotin detection antibody can still be employed with higher specificity but lower sensitivity^6^.

The unique features of the BirA assay have enabled us to conduct the present study, in which we compare the total cellular internalization *and* cytosolic delivery of a variety of protein uptake systems that have been extensively characterized and assessed for cellular delivery, but which had never been quantitatively compared with respect to their ability to achieve cytosolic delivery. Importantly, the current methodology is the only available method that allows the determination of total cellular internalization and cytosolic delivery in parallel, using only a single method and in a generic fashion, thus ensuring a high comparability and broad applicability.

## Results

### Uptake systems investigated

The systems were based on two classical cell-penetrating peptides: *tat* and penetratin^11,12^; three well-studied bacterial toxins: *Pseudomonas* exotoxin A, anthrax toxin and diphtheria toxin^13–15^ and the prototypical supercharged protein, supercharged GFP (scGFP)^3^. As a model cargo, we took the small consensus designed ankyrin repeat protein (DARPin) NI_1_C, which is a non-selected binding protein of intermediate thermodynamic stability with a melting temperature of 60 °C and a size of 11 kDa^16^. The study was designed to investigate the steady-state levels that can be reached by different protein transporters in different cell lines. We focused on a single 20 h time-point, as all vectors will have reached steady-state levels by then (see below).

The uptake systems differ significantly in their uptake mechanisms. At low concentrations, the cell-penetrating peptides *tat* and penetratin predominantly enter cells via distinct endocytic routes and stay in the endolysosomal system, while at higher concentrations, a direct plasma membrane translocation, especially of cationic peptides, becomes more pronounced^17–19^. The prototypical cell-penetrating peptides *tat* and penetratin do not rely on a receptor and are therefore not considered cell-specific^20^.

In contrast, virtually all bacterial toxins do contain a protein domain that is responsible for binding to a specific receptor. In the case of *Pseudomonas* exotoxin A, anthrax toxin and diphtheria toxin, the receptor-binding module can be exchanged for an alternative receptor-binding protein, thereby redirecting the uptake system specifically to a receptor of choice^6,21–23^. The redirected toxin will then be internalized via the endocytic pathway that is associated with the internalization of this particular receptor, before delivering the cargo to the cytosol. It is often unclear how much of the internalized cargo truly reaches the cytosol.

Similar to cell-penetrating peptides, multiple endocytic routes have been found to play a role in the internalization of supercharged proteins, predominantly clathrin-mediated endocytosis and macropinocytosis^24^. Supercharged proteins accumulate mostly in endosomes, as reflected by a punctate fluorescence pattern, even though access of delivered cargo to the cytosol has been reported as well^25^.

### EpCAM as a model receptor

In this study, all receptor-targeted constructs were directed to the epithelial cell adhesion molecule (EpCAM). EpCAM is a cell surface receptor that has often been used for targeted drug delivery, and that is known to lead to rapid internalization after binding^26,27^. EpCAM-mediated internalization has been shown to be very sensitive to the clathrin-mediated endocytosis inhibitor chlorpromazine, thereby identifying clathrin-mediated endocytosis as the main route of entry^28^. This strong reliance on a single uptake route is different from what has been seen for cell-penetrating peptides and supercharged proteins, which can exploit multiple mechanisms at the same time^17,24^. The requirement of available EpCAM for high-affinity binding and a specific route of uptake would suggest that the receptor-mediated internalization would be more active at lower concentrations, but would show signs of saturation. Most importantly, the pivotal role of the surface receptor also makes uptake specific for cells carrying this receptor, rendering import potentially tissue specific.

### Choice of cell lines studied

Here, the uptake was investigated in four cell lines expressing EpCAM, which have all been stably transduced to overexpress the reporter protein BirA: MCF7, SKBR3, HT29 and Flp-In 293 (the latter has been constructed to stably overexpress EpCAM^6^) (Fig. 1b, Supplementary Fig. 1). The complete and rapid biotinylation of cargo molecules was determined for Flp-In 293, MCF7 and SKBR3 cells through overexpression of an HA- and avi-tagged DARPin in the cytosol. After continuous expression of the DARPin for 24 h, cells were lysed and the degree of biotinylation was assessed through a gel retardation assay, where the DARPin is incubated with streptavidin before loading. For all three cell lines, despite high levels of biotinylated DARPins, virtually no unbiotinylated DARPins could be detected (which would remain at its original molecular weight and not co-migrate with streptavidin), indicating that biotinylation is essentially complete under these steady-state conditions.

To investigate how rapidly biotinylation occurs in the cytosol, we also investigated the degree of biotinylation after shorter periods of cytosolic expression. As soon as cytosolic protein could be detected by western blotting (after 8 hours in Flp-In 293 BirA and MCF-BirA cells, probably mostly reflecting the time of DNA uptake, but also transcription and translation), all of the detected protein was fully biotinylated as well (Supplementary Fig. 2). We thus conclude that the biotinylation of avi tags is not rate limiting, and must be rapid. If biotinylation were slow (only occurring >15 min after production), then some level of an unbiotinylated band of the freshly produced DARPin would have been observed in growing cells.

We aimed to provide general insights into the behavior of protein uptake systems, not only in the Flp-In 293 reporter cell line that we previously developed, but also in cancer cell lines with different characteristics. Hence, the main criterion for choosing the cell lines was, next to the fact that they need to express both EpCAM and BirA, that they were sufficiently distinct from each other, so that we cover a wider range of potential similarities and differences. MCF7 and SKBR3 cells are both breast cancer cell lines: MCF7 is classified as a luminal A subtype and SKBR3 is classified as HER2-positive subtype^29^. HT29 is a colorectal adenocarcinoma line and the Flp-In 293 cell line is a human embryonic kidney (HEK) 293-derived cell line modified for ease of creating stable cell lines by recombineering. As mentioned, all cell lines were EpCAM positive, since we wanted to take receptor-mediated uptake and subsequent delivery to the cytosol into consideration. Furthermore, this would also investigate the possibility of being able to obtain cell-type specific cytosolic delivery via the presence or absence of a surface receptor.

In our previous study, we focused on Flp-In 293 cells and to a lesser extent on MCF7 cells^6^, but others have also provided numerous reports on the successful redirection of the bacterial toxins *Pseudomonas* exotoxin A, anthrax toxin and diphtheria toxin towards a variety of protein receptors in all of the four cell lines from the present panel, but comparisons are lacking^14,30–34^. Similarly, cell-penetrating peptides have been extensively studied over the last 20 years, and many reports of *tat* and penetratin as well as fusions thereof have been studied in HEK293, MCF7, SKBR3 and HT29 cells, but also here the results are difficult to compare^35–38^. Supercharged proteins have been less widely studied, though it has been proposed that they can internalize into endosomes in most mammalian cells through interaction with sulfated proteoglycans. Cells that produced only non-sulfated proteoglycans were incapable of efficient uptake of supercharged proteins^3^. Importantly, to this date there exists no study in which comparisons between the different classes of transporters have been reported.

### Protein components of the uptake systems

**Figure 2:**
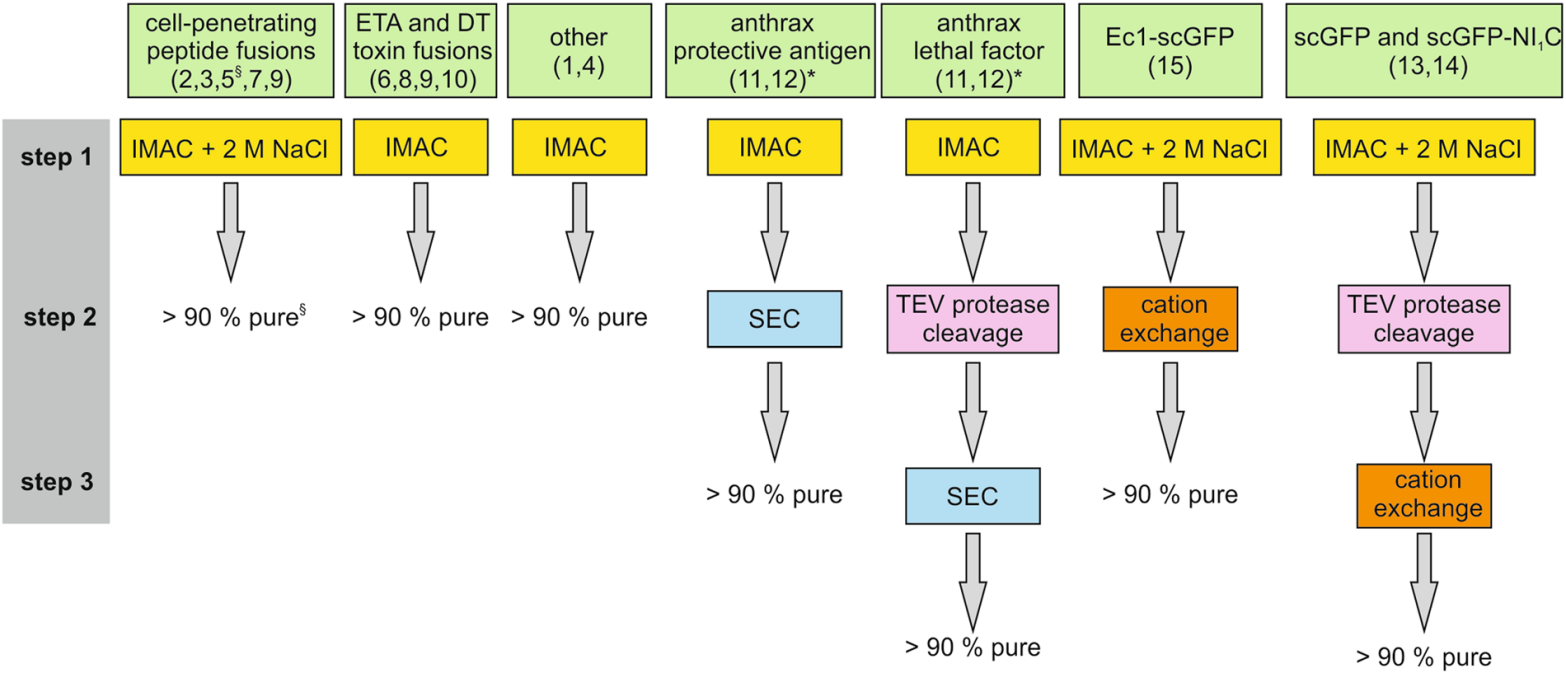
Schematic overview of the purification strategies for the fusion proteins used in uptake experiments. The numbers in the green boxes refer to the numbering system from Fig. 4. § The final purity of #5 was estimated to be ~70%, due to a co-purified degradation fragment (see Fig. 3). * 11 and 12 are binary toxins with two individually produced components. DT, diphtheria toxin; ETA, *Pseudomonas* exotoxin A; IMAC, immobilized metal-ion affinity chromatography; scGFP, supercharged GFP; SEC, size-exclusion chromatography; TEV protease, Tobacco Etch Virus protease.

**Figure 3:**
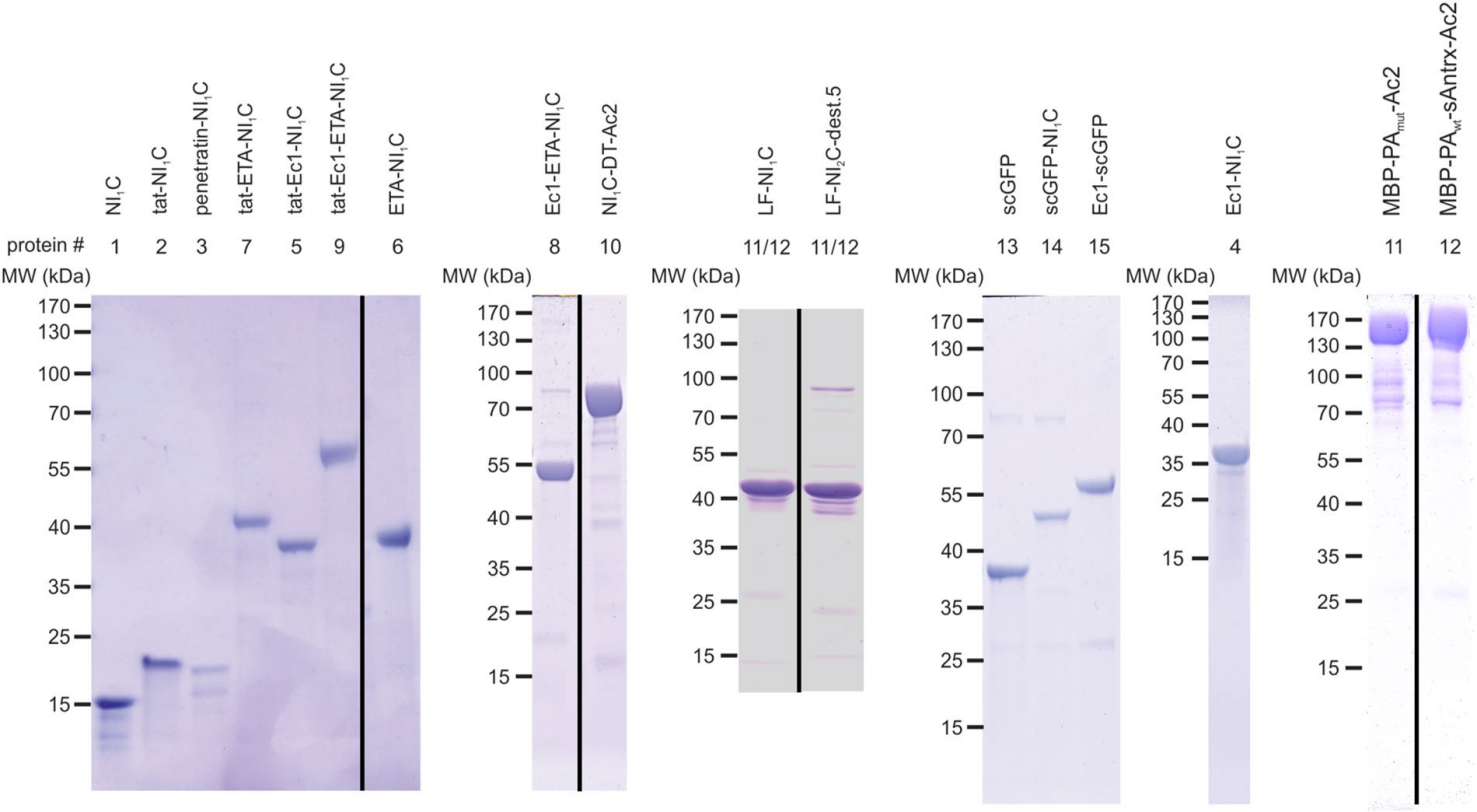
Coomassie blue-stained gels showing the purity of the investigated cargo proteins. The numbering system is according to Fig. 4. Please note that 11 and 12 reflect binary toxins and consist of two proteins. Except for protein #3 (penetratin-NI_1_C), all proteins were considered to have a purity > 90%. To compensate for the lower purity of #3 (~70%), this protein was tested at higher concentrations as well (5 μM). The juxtaposition of lanes from the same gel that were originally non-adjacent is indicated through black vertical lines. Ac2 and Ec1, designed ankyrin repeat proteins recognizing EpCAM; DT, diphtheria toxin; ETA, *Pseudomonas* exotoxin A; LF, lethal factor; MBP, maltose-binding protein; NI_1_C, consensus designed ankyrin repeat protein; PA, protective antigen; sAntrx, soluble anthrax receptor domain; scGFP, supercharged GFP.

**Figure 4:**
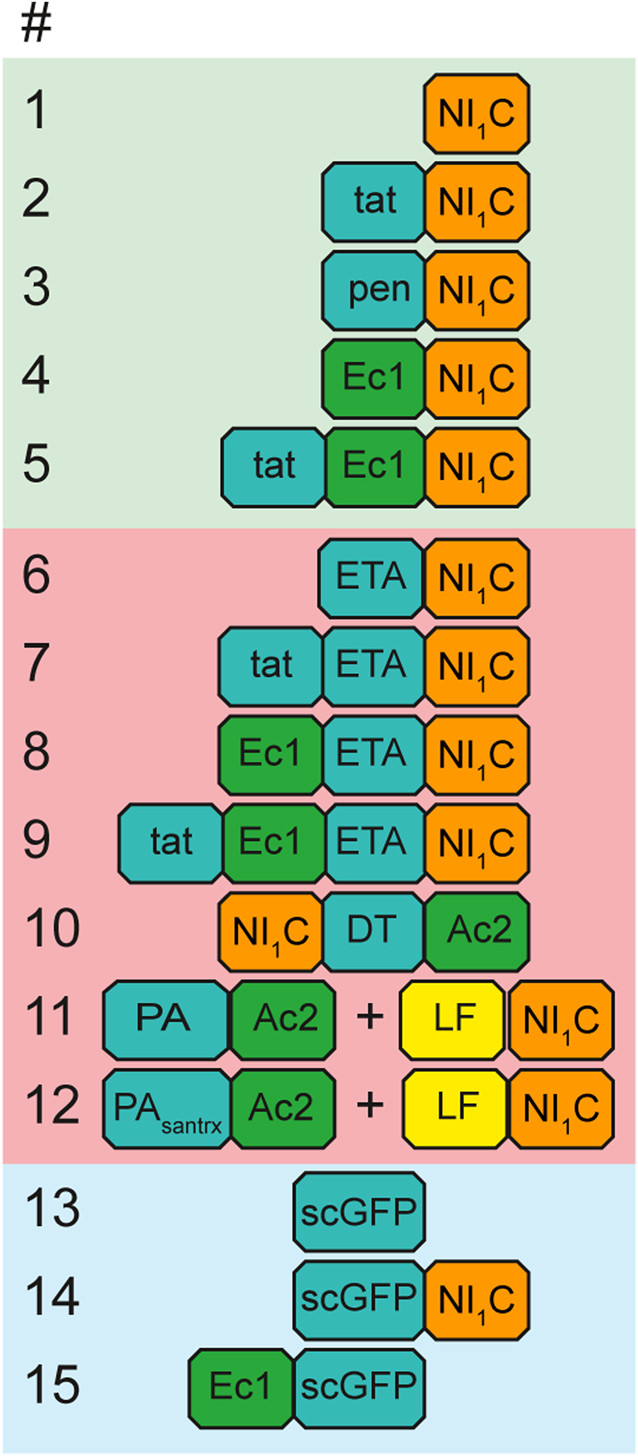
Numbering and block structures showing the modules of the cargo proteins that were assessed in this study. Note that # 11 and # 12 represent delivery systems based on binary toxins and thus encompass two separate components. DT, diphtheria toxin; ETA, *Pseudomonas* exotoxin A; LF, anthrax lethal factor; PA, anthrax protective antigen; pen, penetratin; santrx, soluble anthrax receptor domain; scGFP, supercharged green fluorescent protein.

An overview of the components of the tested protein uptake systems is given in Fig. 1c. Distinct purification strategies were followed for different proteins, depending on the properties of the protein to be purified and on previously established purification protocols. A schematic overview of the purification strategies is given in Fig. 2. The final purity of the proteins was always assessed by Coomassie blue staining and, with the exception of penetratin-NI_1_C, all proteins were considered to have a purity greater than 90% (Fig. 3). The various combinations that were tested are schematically represented in Fig. 4. A representative chromatogram of a cation-exchange chromatography step, used for the purification of supercharged proteins, is given in Supplementary Fig. 3.

### Experimental conditions chosen

**Figure 5:**
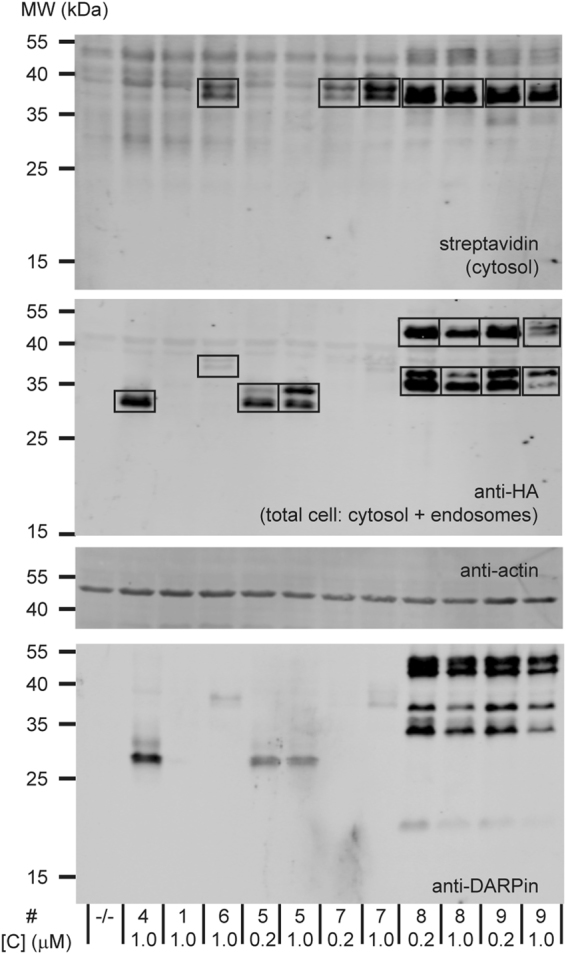
Example western blot of a 4-hour uptake experiment of various protein uptake systems in Flp-In 293 cells. The numbering system is according to Fig. 4. The blot images have been cropped for conciseness. The full-size blots are presented in Supplementary Fig. 5.

**Figure 6:**
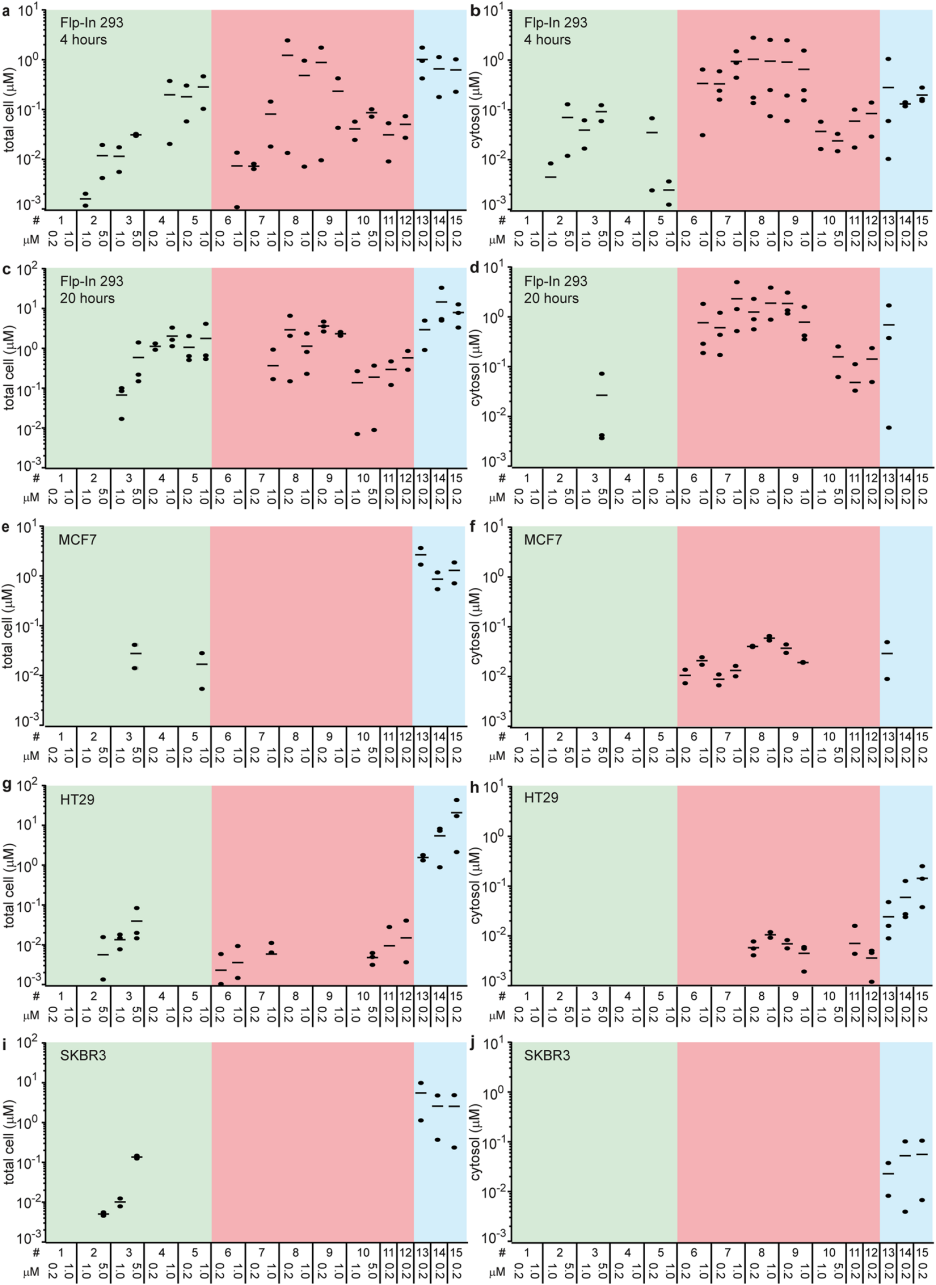
Uptake of various systems in biotin ligase-overexpressing cells. The numbering system is according to Fig. 4. Concentrations are indicated for the respective conditions. LF-cargo fusions were used at 0.2 μM; PA fusions at 20 nM. Quantification of total cellular (**a**,**c**,**e**,**g**,**i**) and cytosolic uptake (**b**,**d**,**f**,**h**,**j**) of 4-hour (Flp-In 293) and 20-hour (all) uptake experiments in the indicated cell lines. Data points reflect absolute concentrations that were present in either the “total cell” or “cytosol” at the time of lysis, calculated using knowledge on the cell number, cell volume and a reference protein, as described in detail in the Methods section. All data points are derived from fully independent experiments. If no data points are shown, no protein could be detected. The horizontal line represents the average. * due to band overlap with an endogenously biotinylated species in the cell line, the cargo LF-NI_1_C was replaced with LF-NI_2_C-dest.5, which has a MW that is 3.4 kDa higher but possesses similar delivery characteristics^6^.

We chose protein concentrations from 200 nM up to 5 μM in the cellular uptake assays, based on the delivery efficiencies of the various uptake systems obtained from preliminary studies. Uptake in Flp-In 293 cells was assessed after 20 h and after 4 h in the presence of proteasome inhibitor (Figs 5 and 6). Uptake in MCF7, HT29 and SKBR3 cells was assessed only after 20 h (Fig. 6). Several combinations of concentrations and constructs that were not tested across all cell lines are given in the Supplementary Table 1 and are specifically referred to in the text.

We chose 20 h as a time-point because the purpose of the experiment was to investigate the steady-state levels that can be reached with these transport systems, rather than the uptake kinetics. In particular the *Pseudomonas* exotoxin A (ETA)-based constructs had previously been shown to have rather slow uptake kinetics^39^, but we reasoned that after 20 h, all transport systems would have reached a steady-state level. To support this hypothesis, we compared the cytosolic uptake of Ec1-ETA(252–412)-NI_1_C (a *Pseudomonas* exotoxin A-based construct) and LF-NI_1_C + PA-Ac2 (an anthrax toxin-based uptake system) as a function of time (Supplementary Fig. 2). Indeed, while the uptake of LF-NI_1_C was somewhat more rapid, reaching maximum levels after 4 h but little decline after 20 h, cytosolic levels of Ec1-ETA(252–412)-NI_1_C only became high after 20 h.

The 4 h time point in Flp-In 293 cells in the presence of proteasome inhibitor was included to investigate delivery to the cytosol without the confounding effect of rapid proteasomal degradation. In the 20 h experiments it was not possible to include proteasome inhibitors due to toxicity of long-term proteasome inhibition.

### Principles of measuring total internalization vs. cytosolic uptake

Western blot data from a representative uptake experiment in Flp-In 293 cells using several of the tested protein uptake systems are given in Fig. 5, with the boxes indicating the bands that were used for determining cellular uptake. Total cellular internalization, reflecting the sum of the uptake in endosomes and cargo delivered to the cytosol, was determined by the HA tag (Fig. 6, *cf*. Figure 1a). It must be emphasized that only the total cellular uptake of fully intact proteins is being detected. The data are given as concentrations in the cell at the time of lysis. We calculated this from the number of cells at the time of lysis, the experimentally estimated volume of the cells and the quantity of the protein detected by western blot, in turn determined with a calibration standard of a fully biotinylated and HA-tagged protein, loaded as a control on each one of the western blots. The protocol is summarized in the Methods section, and more extensively explained in a recent methods paper^10^.

Cytosolic delivery was assessed quantitatively on western blots using fluorescently labeled streptavidin and data are represented as cytosolic concentrations, using the same method as for calculating total cellular internalization (Fig. 6). For ease of comparison, the cytosolic volume was taken to be the same as the total cell volume. Anti-actin and anti-DARPin western blots were included for assessing fair loading and protein degradation, respectively. The polyclonal anti-DARPin serum is less suited for the determination of absolute concentrations, because the quantitative recognition of the epitopes may be DARPin-dependent and because several of the investigated uptake systems contain both a DARPin targeting EpCAM as well as a DARPin cargo. Nevertheless, in situations where degradation of the C-terminal HA tag employed for detection occurs (see below), the use of the DARPin signal can provide important insights into the fate of the transporter and its cargo. In instances where both total cellular internalization and significant cytosolic uptake could be measured, the fraction of intact internalized cargo that reached the cytosol was assessed (Supplementary Table 2).

### Total cellular internalization (i.e., endosomes + cytosol)

Similar uptake patterns could be discerned across multiple cell lines, and we focus first on *total* cellular internalization (i.e., endosomes + cytosol) for the various constructs, without making a distinction about these two different compartments (Fig. 6a,c,e,g,i). Total cellular internalization was extremely high for supercharged GFP (scGFP) variants in all cell lines, presumably because of a very efficient, receptor-independent interaction of the highly positively charged proteins with the negatively charged plasma membrane, especially the negatively charged oligosaccharides exposed to the outside milieu, leading to massive amounts of endocytosis. Already at an external concentration of 200 nM, the average total cellular concentration of scGFP fusions that we determined ranged from 1.7 μM (scGFP) to 23 μM (Ec1-scGFP) in HT29 cells, from 2.9 μM (scGFP-NI_1_C and Ec1-scGFP) to 6.3 μM (scGFP) in SKBR3, from 0.9 μM (scGFP-NI_1_C) to 2.9 μM (scGFP) in MCF7 cells and from 2.9 μM (scGFP) to 15 μM (scGFP-NI_1_C) in Flp-In 293 cells. No clear effect on uptake was observed for the presence of the EpCAM-targeting protein Ec1, though uptake in HT29 tended to be higher for Ec1-scGFP than for scGFP and scGFP-NI_1_C. Notably, in HT29 cells, there was very evident toxicity at 1 μM for all of the supercharged GFP constructs. In the other cell lines, significantly higher uptake levels were observed at 1 μM than at 200 nM, reaching up to an average of 66 μM in Flp-In 293 cells for scGFP (Supplementary Table 1). The higher uptake levels at higher concentrations indicate the absence of any saturatable uptake process for supercharged GFP and fusions thereof.

Total cellular internalization was much lower or even below the detection level for uptake systems based on cell-penetrating peptides and bacterial toxins that were targeted to EpCAM. For cell-penetrating peptide-only fusions (i.e. without a bacterial toxin domain or an EpCAM-targeting DARPin), at an external concentration of 200 nM, total cell concentrations ranged from being undetectable in HT29, SKBR3 and MCF7 cells to 34 nM for penetratin-NI_1_C in Flp-In 293 cells. In general, there was a correlation between higher external concentrations and higher uptake levels, without any evidence of saturation. This is expected for cell-penetrating peptide fusions, and thus similar to the supercharged proteins. At 1 and 5 μM external concentrations, total levels increased to an average of 67 nM and 588 nM, respectively, for penetratin-NI_1_C in Flp-In 293 cells. At 5 μM penetratin-NI_1_C in the medium, a total internal concentration in MCF7 cells of 30 nM was found, in HT29 of 43 nM and in SKBR3 of 149 nM. Tat-NI_1_C was essentially undetectable, except at 5 μM external in SKBR3 cells (5.4 nM) and HT29 cells (6.2 nM). Strikingly, after 4 h in the presence of proteasome inhibitor in Flp-In 293 cells, both tat-NI_1_C and penetratin-NI_1_C were detectable at all external levels tested (200 nM, 1 and 5 μM), giving average values of 14 nM, 16 nM and 118 nM (tat-NI_1_C) and 45 nM, 115 nM and 309 nM (penetratin-NI_1_C) (Fig. 6a; Supplementary Table 1). Adding the EpCAM binder Ec1 to the fusion between the cell-penetrating peptide *tat* and NI_1_C only yielded a benefit in MCF7 cells, where at 1 μM the fusion tat-Ec1-NI_1_C, but not tat-NI_1_C or Ec1-NI_1_C gave a detectable signal (18 nM). Besides the scGFP fusions, where uptake is independent of the EpCAM-targeting DARPin Ec1, this was the only occurrence where an Ec1 fusion was detected in MCF7 cells.

Fusion proteins containing bacterial toxins and the EpCAM-targeting domain and/or the cell-penetrating peptide *tat* performed well in Flp-In 293 cells with respect to total internalization: the Ec1-DARPin ETA fusion gave better results than the tat-ETA-NI_1_C fusion, as the latter only reached detectable levels at 1 μM. Notably, the EpCAM-DARPin Ec1 fused to NI_1_C was also detected at high levels, indicating high levels of EpCAM. In HT29 cells, bacterial toxin-domain systems performed less well, but were in several instances still detectable via the HA tag: a fusion between *tat* and ETA-NI_1_C and fusions between diphtheria toxin and anthrax toxin systems and the EpCAM binder Ac2 were still detected (Fig. 6g). This was not the case in MCF7 or SKBR3 cells, again indicating either only moderate EpCAM levels or a high proteolytic activity causing cleavage of the HA tag. In contrast to cell-penetrating peptide- and scGFP-fusions, for toxin-based constructs, total internalization was not or not much higher at 1 μM than at 200 nM. Also in view of the high affinity of the EpCAM-targeting DARPins (61 pM for Ec1 and 1.2 nM for Ac2^40^), this indicates an almost full saturation of the receptor-mediated endocytic internalization at 200 nM (Fig. 6a,c,g).

### Delivery to the Cytosol

The quantity of greatest interest, delivery to the cytosol, followed a strikingly different pattern compared to total internalization (Fig. 6b,d,f,h,j). For the fraction of the internalized cargo that reached the cytosol (Supplementary Table 2), the calculations yielded values higher than 100% in multiple instances, effectively meaning that more biotinylated protein was detected than HA-tagged protein. Furthermore, there were a number of cases where HA-tagged proteins could not be detected, whereas biotinylated proteins could. Since anti-DARPin signals were detected in such instances (Supplementary Fig. 4 and Supplementary Table 3), this is presumably due to cleavage of the HA tag, as described before^6,41^. The higher sensitivity of the biotin-streptavidin interaction, as compared to the HA-antibody interaction, may play a role as well^6^. A possible cleavage of the HA tag is not an inherent limitation of the biotin ligase assay, as the HA tag can in principle be exchanged for any other tag. Since in the present study the HA tag was positioned behind the avi tag, its cleavage is certainly possible, and would lead to apparent fractions of cytosolic cargo that exceed 100%. To avoid confusion, we listed the apparent fraction for these cases as >100%. To provide further insight into the delivery process, we estimated the total cellular internalization in some instances additionally through the anti-DARPin antibody. Quantitation in this case is less straightforward since the antibody is polyclonal, and in some instances two DARPins reside in the same protein transport system.

The control construct that consisted of the EpCAM-targeting moiety Ec1 directly fused to the cargo NI_1_C, not containing any transport domain, showed good receptor-mediated internalization into endosomes in Flp-In 293 cells, but no delivery into the cytosol, as expected. Both transporters based on *Pseudomonas* exotoxin A (ETA) and anthrax toxin yielded detectable levels in HT29 cells (Fig. 6h) and performed well in Flp-In 293 (Fig. 6b,d), with average levels of 5.8 nM for Ec1-ETA-NI_1_C and 7.1 nM for the anthrax toxin-based construct (#12) in HT29 cells, and 1.4 μM and 49 nM for the same constructs in Flp-In 293 cells, all at an external concentration of 200 nM. The fraction delivered to the cytosol would be calculated as 52% to more than 100% for the anthrax toxin-based construct and appeared >100% for Ec1-ETA-NI_1_C, presumably all due to HA tag cleavage, as explained before. Also here, the higher levels in Flp-In 293 cells are likely a consequence of the high levels of EpCAM, as well as the high efficiency of cytosolic delivery of the ETA construct in Flp-In 293 cells. In this cell line, uptake was also assessed after 4 h in the presence of proteasome inhibitor. Levels of Ec1-ETA-NI_1_C and the anthrax toxin-based construct were similar to those at 20 h, with average values of 1.0 μM and 85 nM, respectively, indicating no major effects of proteasomal degradation for these transporters. The apparent fraction delivered to the cytosol appeared again >100% for the ETA-based constructs across many conditions, and ranged from 11% to 28% for the anthrax-based constructs in Flp-In 293 cells.

In MCF7 cells, only the toxin-based systems that incorporated an ETA translocation domain resulted in a good cytosolic delivery. Also in this cell line, the highest delivery was observed for Ec1-ETA-NI_1_C with average levels of 40 nM and 59 nM at 200 nM and 1.0 μM, respectively. No HA-tagged proteins were detected under these conditions but good anti-DARPin signals were detected in this cell line, indicating HA tag cleavage. Since in Flp-In 293 cells good signals for both HA and anti-DARPin signals were observed after 20 h for similar constructs, this indicates that the rate of HA tag cleavage is mainly cell-specific, and not construct-specific (Supplementary Table 4). Furthermore, no increase in degradation over time was observed in Flp-In 293 cells, when ratios after 4 and 20 h were compared (Supplementary Table 4). Importantly, the fact that HA tag cleavage is a cell-specific phenomenon means that valid comparisons of constructs within a single cell line can be done.

It is in principle possible to perform a similar calculation of total cellular uptake using anti-DARPin signals, of which the results from MCF7 cells for three proteins are given in Supplementary Table 3, even though estimations should be interpreted with caution because of the polyclonal nature of the anti-DARPin signal. For the diphtheria toxin-based constructs, where no cytosolic uptake was detected, total uptake values varied between 11 and 40 nM at external concentrations of 200 nM and 5 μM, respectively. For the two ETA-based constructs, total uptake levels varied between 30 and 110 nM, and were similar to what was observed for the cytosolic uptake, again confirming that a very large fraction of internalized ETA protein reaches the cytosol in MCF7 cells.

In MCF7 cells, the *tat* functionality did not lead to any cytosolic uptake, remaining the same as the control devoid of any uptake system (ETA-NI_1_C), and *tat* even decreased the total cellular uptake in the presence of the EpCAM binder Ec1, as assessed via the anti-DARPin serum signal. Surprisingly, in SKBR3 cells, none of the cell-penetrating peptide fusions nor any toxin domain fusions yielded detectable cytosolic levels. The latter could be either due to a high proteolytic activity towards the tags that have to be detected, to rather modest levels of EpCAM, or to a slower internalization rate of EpCAM in this particular cell line.

The diphtheria-based construct did not achieve efficient transport to the cytosol of NI_1_C in HT29, MCF7 or SKBR3 cells, even though low amounts of cytosolic delivery could be detected in Flp-In 293 cells, both after 20 h (158 nM at 5 μΜ) and after 4 h (24 nM at 5 μM external concentration) in the presence of proteasome inhibition (Fig. 6b,d).

Cytosolic delivery of cell-penetrating peptide fusions, without the presence of an ETA domain, was undetectable in HT29, MCF7 and SKBR3 cells. Only in Flp-In 293 cells could the penetratin-NI_1_C fusion be detected at the highest levels tested, 5 μM. Here, an average cytosolic concentration of 38 nM was detected, estimated to indicate that between 2 and 5% of internalized cargo has reached the cytosol. In contrast to what was observed for toxin domain fusions, fusions between NI_1_C and cell-penetrating peptides performed markedly better after 4 h in the presence of proteasome inhibitor as compared to after 20 h, with average levels of 71 and 92 nM for tat-NI_1_C and penetratin-NI_1_C at 5 μM, respectively, but also giving detectable levels for both fusions at 1 μM (4.5 nM – 39 nM) and 200 nM (7.8 nM – 25 nM). This indicates that at least part of the low efficiency observed may be due to a poor cytosolic stability.

Despite the massive total cellular internalization, scGFP-based constructs showed little delivery to the cytosol or none at all, indicating a poor endosomal escape efficiency. In MCF7 cells, at 200 nM only scGFP without cargo was detected, but this was at the comparably low level of 21 nM, which corresponded to only 0.2–0.4% delivery to the cytosol (Fig. 6f). Levels in HT29 were similarly low, with average values of 16 nM at an external concentration of 200 nM (Fig. 6i), with calculated fractions of cytosolically delivered cargo between 0.5% and 1.1%. Also in Flp-In cells and SKBR3 cells, the cytosolic fractions were low and ranged from 0.7% – 7.5% (Flp-In 293) and 0.3% – 0.7% (SKBR3). These numbers are in stark contrast to what was observed for toxin-based protein transporters, where the apparent delivered cargo often was higher than 100%. It must be noted, however, that in the case of SKBR3 cells, cytosolic delivery could only be detected with scGFP, and not with cell-penetrating peptide or toxin domain fusions. Normally, scGFP would not be the sole cargo itself but would to be used to deliver another protein, and thus fusion proteins need to be investigated. In MCF7 and Flp-In 293 cells, coupling of scGFP to either a cargo (NI_1_C) or a receptor-targeting agent (Ec1), both adding negative charges to the construct, negatively influenced the ability to escape the cytosol (Fig. 6b,d,f), even though this was not evident in HT29 and SKBR3 cells (Fig. 6h,j).

Through the use of an HA-tagged and biotinylated recombinant protein as a reference and knowledge about the cell number and volume, we could estimate absolute concentrations present in the cytosol. Determined levels ranged from low nanomolar to low micromolar, with especially high concentrations found in the Flp-In 293 cells that artificially overexpressed EpCAM. In the present study, values were expressed as absolute cellular concentrations, but a conversion to molecules per cell using the cell volumes that were measured in this study (2.9 pL for HT29 cells, 7.2 pL for MCF7 cells, 2.2 pL for Flp-In 293 cells and 4.4 pL for SKBR3 cells) are straightforward, leading to values ranging from the lower detection limits of ~38,000 molecules/cell (8.8 nM) up to ~258,000 (59 nM) in MCF7 cells, from ~4,500 (3.6 nM) to ~3,280,000 (2.7 μM) in Flp-In 293 cells, from ~7,800 (4.5 nM) to ~247,000 (143 nM) in HT29 cells and from ~63,000 (23 nM) to ~613,000 molecules/cell (223 nM) in SKBR3 cells.

## Discussion

The sensitive, generic and objective nature of the biotin ligase assay has allowed the acquisition of data that clearly demonstrate that endosomal uptake does not correlate with delivery into the cytosol. The assay thus differentiates the different systems regarding both quantities. Our data show that endosomal uptake can be achieved with cell-penetrating peptides, with a receptor-targeting moiety or via supercharged proteins. In contrast, endosomal escape can be efficiently achieved only with bacterial toxin-based protein transporters, which appear to have evolved for this purpose, but not or only poorly with fusions to cell-penetrating peptides or supercharged proteins (Fig. 6). Moreover, CPP and scGFP lack cell specificity in endocytosis. Therefore, to bring CPP or scGFP fusions and their cargoes efficiently into the cytosol, further modifications would be necessary for them to be transported into the cytosol from the endosomes. Particularly for cell-penetrating peptides, various strategies are currently being investigated to achieve this, e.g. incorporation into branched systems or the combination with endosome-disrupting peptides or alternative endosome-disrupting moieties^42,43^, with novel strategies continuously being explored^44,45^. Also for supercharged proteins, the lack of cytosolic delivery has been previously been recognized and a peptide was recently identified that could enhance the endosomal escape severalfold^46^. Nonetheless, their lack of specificity for the cell type is an intrinsic feature of this charge type interaction, potentially limiting their *in vivo* use.

Our investigations were performed in a panel of four different cell lines, which were chosen to reflect the variety in how cells deal with uptake systems. Despite a number general trends, we did observe large differences between the cell lines. Factors that are likely to affect the differences are many, and include the amount of EpCAM present on the cell surface, their endolysosomal protease activity, the amount of furin present (needed for processing of the bacterial transport systems) and the degradative activities present in the cytosol (limiting the steady-state levels), of both the entire cargo and/or the HA tag specifically. The large variety highlights the challenges in developing a cytosolic protein delivery approach that works in many cell lines, including primary cell lines.

Whether protein-protein interactions can be inhibited through delivered binding proteins using the systems tested here will depend on the protein in question, as protein abundances are known to vary widely over many orders of magnitudes, from as low as 125 molecules per cell to >1 million per cell^47^. For many targets, the levels reached would be sufficient, as are the achievable binding affinities. It should be pointed out, however, that for most applications delivery will have to take place in more challenging environments, where delivery will be lower, implying that further improvements for all systems are still highly desirable.

In this study, we looked at steady-state levels that the transport systems might reach, which is of course of key importance when protein delivery is used as a tool either for fundamental research or for therapy. Nevertheless, a limitation of this study is that we only looked at a single time-point. It is known that transporters have different kinetics (cf. Supplementary Fig. 2), with the entry of cell-penetrating peptides and supercharged proteins particularly rapid^3,48^, whereas internalization via bacterial toxin-derived components can be slower, in particular for *Pseudomonas* exotoxin A-based systems^39^. It is therefore possible that due to (extracellular) degradation activity, in particular of unstructured cell-penetrating peptides, the levels for cell-penetrating have been lower than what would have been observed after shorter incubation times.

In conclusion, for many years, endosomal escape has been named as a major bottleneck in the delivery of macromolecular cargo such as proteins to the cytosol^49,50^. Here, we confirm and extend this notion by providing objective quantitative insights into this process for a small model cargo using a method that can be transferred to any cargo of interest, and we contrast this with uptake via protein toxins which have evolved for this purpose.

## Methods

### Cell culture

Flp-In 293 cells overexpressing EpCAM and BirA and HT29-BirA cells were maintained in DMEM supplemented with 10% fetal calf serum (FCS; Amimed). 1000 μg/mL G418 was included for HT29-BirA cells. MCF7-BirA cells were maintained in a HAM/DMEM mix (50:50) supplemented with 10% FCS and 400 μg/mL G418. SKBR3-BirA cells were maintained in RPMI supplemented with 10% FCS and 200 μg/mL G418. All media were supplemented with 100 IU/mL penicillin and 100 μg/mL streptomycin (Sigma). All cell lines were tested negative for mycoplasma contamination.

### Generation of stable BirA cell lines

Flp-In 293 cells were purchased from Invitrogen, HT29 cells from DSMZ (Deutsche Sammlung von Mikroorganismen and Zellkulturen) and SKBR3 and MCF7 cells were purchased from ATCC (American Type Culture Collection). All cell lines were obtained in their original state, i.e. without genetic modifications. The generation of the Flp-In 293 cell line stably overexpressing EpCAM and BirA has been described before^6^. For MCF7, SKBR3 and HT29 stable cell line generation, a plasmid containing a cytomegalovirus promoter-driven *birA*, followed by an encephalomyocarditis virus (EMCV) internal ribosomal entry site (IRES) and neomycin resistance gene, was constructed by cloning the complete *E*. *coli birA* gene in the pCMV-EMCV-NEO vector (kind gift of Dr. J. Piehler, University of Osnabrück). A clonal dilution was made and single-cell-derived colonies were obtained in the presence of 400 μg/mL (MCF7) or 200 μg/mL (SKBR3) G418. Stable pools, continuously under selection with 1000 μg/mL G418, were obtained with HT29 cells. The activity of the biotin ligase-expressing cells was confirmed via the cytosolic overexpression of a DARPin or of a fusion of eGFP to the consensus DARPin NI_3_C^16^, in either case containing both an HA tag (YPYDVPDYA) and an avi tag (GLNDIFEAQKIEWHE)^6^ (Fig. 1b, Supplementary Fig. 1). The vector pcDNA3.1( + ) was used in both instances.

### Biotin ligase uptake assay

#### Conditions during cellular incubations

Uptake experiments were exclusively performed with cell lines stably overexpressing biotin ligase. Briefly, cells were seeded in 24-well plates: 300,000 cells per well for Flp-In 293 and HT29 cells and 150,000 for MCF7 and SKBR3. To check for biotinylation of the avi tag by the overexpressed biotin ligase, one well was transfected in each experiment with the cytosolically expressed non-selected DARPin E3_5, containing both an avi and an HA tag. For MCF7 and HT29 cells, the consensus DARPin NI_3_C fused to eGFP was used (avi-HA-NI_3_C-eGFP), also containing both an avi and HA tag, as the naked E3_5 DARPin resulted in only comparably low expression levels after 24 hours. Transfections were performed using TransIT-293 (Flp-In 293), TransIT-X2 (HT29) or TransIT-LT1 (MCF7 and SKBR3) using the manufacturer’s protocol (Mirus).

Proteins were diluted to the indicated concentrations in the same complete medium that was used for cultivating the cells, supplemented with 0.1 mM biotin (final concentration). When performing 4-hour incubations, the proteasome inhibitor MG-132 (Calbiochem, Merck Millipore) was added to a concentration of 50 μM in the medium. 400 μL of the prepared protein in complete medium was added to each well.

#### Lysis procedure

After the indicated incubation time, the medium was removed from the wells. All cells were washed once with 500 μL Dulbecco’s PBS (Sigma), except Flp-In 293 cells, which adhere only lightly and were not washed. Cells were detached by adding 100 μL trypsin-EDTA solution (Sigma) and incubated for approximately 5 min at 37 °C. Subsequently, 800 μl fresh complete medium was added to the cells to dilute the trypsin. The mixture was then transferred into 1.5 mL microcentrifuge tubes. Cells were centrifuged at 300 × g for 3 min and resuspended in 800 μL Dulbecco’s PBS after removing the supernatant. The centrifugation step was repeated and excessive PBS was removed. Finally, the cells were lysed by resuspending the cell pellets in 50 μL hot (96 °C) SDS lysis buffer that was supplemented with 20 μM of a stabilized avi tag peptide^6^ and further heating the samples for 8 min at 96 °C. The cells that were transfected with the control DARPin or DARPin-eGFP construct containing avi and HA tags were lysed with the same protocol. Before western blotting, the lysate was divided and diluted fourfold in a non-denaturing, non-reducing buffer (0.5 M Tris-HCl, pH 6.8, 20% (v/v) glycerol, 0.02% (w/v) bromophenol blue), either containing streptavidin (concentration: 50 μg/mL) or not, and incubated for 2 h at 4 °C. Samples were stored at −20 °C until analysis by western blotting.

#### Western blotting

Western blotting was performed essentially as described previously^6^. Briefly, Immobilon-FL PVDF membranes (Merck Millipore) were pre-activated with MeOH and used for wet blotting for 1 h at 100 V. Blocking was accomplished with casein blocking buffer (Sigma) for 30 min at room temperature while shaking on a roller shaker. Then, the primary antibody solution in PBS with 0.1% (v/v) Tween-20 and 1 × casein blocking buffer (antibody incubation buffer) was applied on the blot and incubated for 1 h at room temperature while shaking on the roller shaker. The primary antibodies used were streptavidin-IRDye 680LT (LI-COR Biosciences), polyclonal rabbit anti-HA (H6908, Sigma-Aldrich), mouse anti-actin (ab8224, Abcam) and a home-made rabbit anti-DARPin serum. Secondary antibodies were goat anti-rabbit Alexa Fluor 680 (Invitrogen), donkey anti-mouse IRDye800 CW (LI-COR Biosciences) and goat anti-rabbit IRDye800 CW (LI-COR Biosciences). When incubating with the fluorescently labelled streptavidin (IRDye 680LT), also 0.1% (w/v) SDS was included to reduce non-specific binding of streptavidin. Blots were either incubated with streptavidin-IRDye 680LT and anti-DARPin serum together, or with the anti-HA and anti-actin antibodies together. Detection was accomplished using an Odyssey 9120 imaging system (LI-COR Biosciences).

#### Data analysis

Bands corresponding to the expected molecular weights were quantified using Image Studio Lite software for determining total cell (anti-HA) and for cytosolic (IRDye 680LT-labeled streptavidin) concentrations. This imaging method gives a highly linear signal range, which has been confirmed before^6^. Identical image acquisition settings were used for distinct blots within a single experiment. In some cases where excessive differences in signal intensities were observed (i.e. > 10,000 times higher in intensity for scGFP vs bacterial toxin constructs in total cell uptake), additional images were acquired at a lower intensity to avoid oversaturation issues. A loading control for calibration purposes was also present in these instances.

Bands were only quantified when they could be clearly identified as a separate band by eye and when they were not observed in an untreated sample (e.g. unspecific bands), which was included on every western blot. All conditions were investigated in two or three fully independent experiments (i.e. biological replicates). All individual data points are shown for conditions where the band could be detected in at least two fully independent experiments.

To correct for differences in signal acquisition between blots and experiments, 0.50 pmol of a fully biotinylated and HA-tagged protein was loaded on each blot as a calibration control (either Ec1-ETA(252–608)-NI_3_C, Ec1-ETA(252–412)-NI_1_C or LF-NI_3_C). To correct for the staining intensity with streptavidin-LT680 between blots in a single experiment, the strongest unspecific signal (which is independent of the cargo protein) was quantified in the first 10 wells containing cell lysate of each blot. This normalization protocol was chosen as it proved to be more accurate than using the streptavidin signal from the fully biotinylated protein. Signals from the HA-tagged and biotinylated reference protein (reflecting 0.5 pmol protein) were then used to calculate back the amount of HA-tagged and biotinylated cargo protein in the lysate. Since the number and volume of the cells in the 24-well plate was determined after each experiment with the CASY Counter (Roche), which calculates the cell volume based on electric current exclusions and pulse area analysis, the amount of protein in the lysate could be converted to the actual concentration in the cell or cytosol. An equal volume for cell and cytosol was assumed and the contribution of the nucleus was left out, which will lead to a moderate underestimation of the actual cytosolic concentration, but enhances the comparability of the results of “total cell” and “cytosol”, as it compares the number of molecules per cell. Note, however, that the actual volume of the endosomes is much smaller than the cell volume, such that the local concentration of a protein exclusively located in endosomes will be significantly underestimated.

### Cloning, protein expression, protein purification and *in vitro* biotinylation

For details on the abovementioned procedures, please see the Supplementary Methods section.

## Electronic supplementary material

Supplementary Figures, Tables and Methods
