# Supplementary material for: A quantitative comparison of cytosolic delivery via different protein uptake systems
Source: Sci Rep. 2017 Oct 16;7:13194. doi: 10.1038/s41598-017-13469-y (PMC5643320; doi:10.1038/s41598-017-13469-y)
Supplement: Supplementary file 1 — Supplementary Figures, Tables and Methods [file 41598_2017_13469_MOESM1_ESM.pdf]

Supplementary Information

for

A quantitative comparison of cytosolic delivery via different protein uptake systems

Wouter P.R. Verdurmen, Marigona Mazlami

and Andreas Plückthun*

University of Zurich, Dept. of Biochemistry, Zurich, Switzerland

Supplementary Methods

Cloning

All constructs were cloned with an N-terminal MRGS-His_6, to enable an immobilized metal-ion affinity chromatography (IMAC) purification step, and contained both an HA tag and an avi tag for use in the BirA assay. Cloning steps for all constructs were performed either with the *E. coli* strain NEB5α or the *E. coli* strain XL1-blue. The consensus DARPin NI_1C[1] and Ec1-NI_1C, a fusion of NI_1C to the EpCAM-targeting designed ankyrin repeat protein (DARPin) Ec1, were both assembled in the vector pDST067 via a PCR amplification step. ETA(252-412)-NI_1C, containing the translocation domain of *Pseudomonas aeruginosa* exotoxin A (ETA), was constructed via PCR from the vector pQIq containing Ec1-ETA(252-412)-NI_1C that has been described previously[2]. The diphtheria toxin (DT) fusions NI_1C-DT-Ac2 was prepared by cloning the DNA sequence coding for amino acid residues 1-389 of diphtheria toxin from *Corynebacterium diphtheriae,* including the inactivating mutation G79D, between NI_1C and Ac2 in pQIq. The amino acid sequence 1-389 encompasses both the inactivated catalytic domain and the T-domain responsible for protein translocation, but not the original receptor-binding domain. The diphtheria toxin gene fragment was purchased as a gene from Genscript. DNA blocks containing the *tat* and penetratin sequences were ordered from Integrated DNA Technologies. PCR amplicons prepared from these DNA blocks containing the required restriction sites were used for the construction of penetratin-NI_1C, tat-NI_1C, tat-Ec1-NI_1C, tat-ETA(252-412)-NI_1C and tat-Ec1-ETA(252-412)-NI_1C in pQIq[2]. The DNA sequence of supercharged GFP (scGFP) (+36) was based on a published protein sequence[3] and ordered as a DNA block from Geneart (Life Technologies). It was inserted into the pQIq vector between an N-terminal MRGS-His_6 and C-terminal avi and HA sequences, resulting in the scGFP construct. The model cargo DARPin NI_1C was introduced C-terminally by PCR. The scGFP and scGFP-NI_1C stretches were transferred into a pQIq vector with an N-terminal Ec1 fusion, yielding Ec1-scGFP and Ec1-scGFP-NI_1C. A Tobacco Etch Virus (TEV) protease cleavage site (ENLYQFG) was introduced by PCR between Ec1 and scGFP to produce Ec1-scGFP-NI_1C and Ec1-scGFP variants that yielded scGFP-NI_1C and scGFP after TEV protease cleavage (see below for further details). The cloning of PA-Ac2 and LF1-254-NI_1C and LF1-

254-NI_2C-dest has been described before[2]. PA_{sAntrx}-Ac2 was prepared by genetic fusion of the soluble part of anthrax toxin receptor 2 (residues 40-217), bearing the mutation C175A, to wild-type protective antigen by a $(G_4S)_5$ linker with a length of ~88 Å, and followed by the EpCAM-targeting DARPin Ac2. A more extensive description of design features and the characterization of this particular protective antigen variant will be published elsewhere.

Protein expression

E. coli strain BL21 (DE3) was transformed with all plasmids for expression, except those that contained an ETA translocation domain (252-412), for which the *E. coli* strain Origami B (DE3) was used. A single clone was picked on the next day and used for the inoculation of 50 mL overnight cultures in lysogeny broth (LB) medium containing 1% (w/v) D-glucose and supplemented with 100 µg/mL ampicillin (BL21 (DE3)) or 100 µg/mL ampicillin and 15 µg/mL kanamycin (Origami B (DE3)). On the next day, the overnight culture was used to inoculate 1 L of terrific broth (TB) medium (OD_{600} after inoculation ~ 0.1) for expression, supplemented with 0.8% (w/v) D-glucose and antibiotics. The cultures were grown at 37°C until an OD_{600} of 0.6 to 0.8 was reached, at which point expression was induced with 500 µM isopropyl-*β*-thiogalactoside (IPTG) and the temperature was lowered to 25°C. After 4 h of expression, the cultures were centrifuged, shock-frozen and stored at -80°C until purification.

Protein purification

A schematic overview of the purification strategies of the various proteins is given in **Fig. 2**. All proteins, except fusions containing a highly positively charged moiety such as *tat*, penetratin or scGFP, were purified as described previously[2]. For constructs containing such a highly positively charged moiety, the salt concentration in all buffers was increased to 2 M NaCl in order to prevent co-precipitation with or co-purification of negatively charged cellular components. Besides changing the salt concentration from 150 mM to 2 M, the procedures remained unmodified. Briefly, cell pellets obtained from 1 L cultures were resuspended in 50 mL cold 2-[4-(2-hydroxyethyl)piperazin-1-yl]ethanesulfonic acid (HEPES)-buffered saline wash (HBS-W) buffer (50 mM HEPES, 150 mM or 2 M NaCl, 1 mM $MgCl_2$, 20 mM imidazole, 0.1 mM 4-(2-aminoethyl)benzenesulfonyl fluoride hydrochloride

(AEBSF), 1 µM leupeptin and 1.4 µM pepstatin-A, pH 8.0). Pellets were lysed with a commercial homogenizer (SLM Aminco Thermo French Press or Constant Systems) or an in-house designed homogenizer in 1 to 3 cycles. The cell debris was removed by centrifugation at 4°C for 40 min at 28,000 × g, and subsequent filtration through 0.22 µm pore-sized membranes (Merck Millipore). A benchtop IMAC procedure was performed as described[2]. The protein purity was assessed by staining of SDS-polyacrylamide gels with Coomassie blue dye (**Fig. 3**) and the OD_{260}/OD_{280} ratio as determined with a NanoDrop® 2000 spectrophotometer (Thermo Fisher Scientific). All constructs were found to be over 90% pure as estimated by Coomassie blue staining, except for penetratin (~ 70 % pure) and scGFP constructs. scGFP constructs were further purified by cation exchange, as described below.

TEV Protease Cleavage: Two scGFP-based constructs, scGFP and scGFP-NI_1C, were expressed with an N-terminal Ec1-DARPin followed by a TEV protease cleavage site, ENLYQFG, as the expression without the solubility-enhancing N-terminal DARPin was unsuccessful. The purification of TEV protease has been described elsewhere[2]. TEV protease cleavage was accomplished overnight at 4°C at a 10:1 (w/w) ratio of protein to TEV protease in a sodium acetate – acetic acid buffer, pH 5.6, containing 0.2 or 0.5 M NaCl and supplemented with 0.5 mM EDTA and 1 mM DTT. Success of TEV cleavage was checked by Coomassie blue-staining of SDS polyacrylamide gels and was typically complete.

Cation exchange chromatography: Constructs based on scGFP were less than 90% pure as assessed by Coomassie blue staining and were further purified by cation exchange chromatography. IMAC-purified and TEV protease-cleaved scGFP-based constructs were applied to the strong cation-exchanger column Mono S 5/50 GL (GE Healthcare) using an ÄKTA pure or ÄKTA explorer system (GE Healthcare). The column was equilibrated with 10 column volumes of PBS buffer containing 0.5 M NaCl (pH 7.4). Protein samples were diluted to 0.5 M NaCl in PBS prior to directly loading them onto the column, which was followed by washing with 20 column volumes with PBS containing 0.5 M NaCl. Elution was carried out with a linear gradient of 0.5 M NaCl to 2 M NaCl (all in PBS) over 10 column volumes (**Supplementary Fig. 3**). Protein fractions were combined and dialyzed back to PBS with 2 M NaCl.

Removal of proteins already biotinylated in the production host E. coli: The fraction of expressed protein that was biotinylated due to the activity of native biotin ligase in *E. coli* was removed from all proteins using a streptavidin resin (Genscript) as described previously[2]. For penetratin-NI_1C and tat-Ec1-ETA-NI_1C, the published procedure was unsatisfactory. We hypothesized that a small biotinylated fraction remained, due to the inaccessibility to streptavidin of biotinylated avi tags residing within soluble protein aggregates. Addition of 1 M guanidinium chloride led to a complete removal of any biotinylated species, presumably through reversibly dissolving these soluble protein aggregates. The guanidinium chloride was dialyzed away after removal of the biotinylated fraction. Finally, proteins were concentrated using Amicon® Ultra Centrifugal Filters (Merck Millipore) with either 3 kDa or 10 kDa cut-offs, depending on the size of the protein. The proteins were concentrated to > 40 µM such that during the incubation of protein with cells, the concentration of PBS with 2 M NaCl would be diluted again: it was lower than 12.5% at 5 µM protein, lower than 2.5% at 1 µM protein, and lower than 0.5% at 200 nM protein. Aliquots were shock-frozen in liquid N_2 and stored at -80°C.

***In vitro* biotinylation**

For Ec1-ETA(252–608)-NI_3C, Ec1-ETA(252–412)-NI_1C and LF-NI_3C biotinylation was carried out *in vitro*[2]. The reaction was done in a volume of 50 µL with a protein concentration of 40 µM in a buffer containing 50 mM bicine, 50 mM NaCl, 10 mM ATP, 10 mM $MgAc_2$, 50 µM biotin and 2.5 µg biotin ligase (kind gift of N. Stefan). For this purpose, the proteins were first buffer-exchanged from PBS into 0.5 M bicine, 0.5 M NaCl, pH 8.3. After mixing the remaining components together, the mixture was incubated for 3 hours at 30 °C and then buffer-exchanged twice against PBS using Zeba Spin Columns (Pierce Biotechnology, Inc.). Since the OD_{260}/OD_{280} ratios were still high (~0.80 to 1.50), the samples were dialyzed against PBS overnight and concentrated using 30 kDa cut-off Amicon® Ultra Centrifugal Filters (Merck Millipore), which yielded acceptable OD_{260}/OD_{280} ratios for all proteins (0.60-0.80).

Supplementary Figures

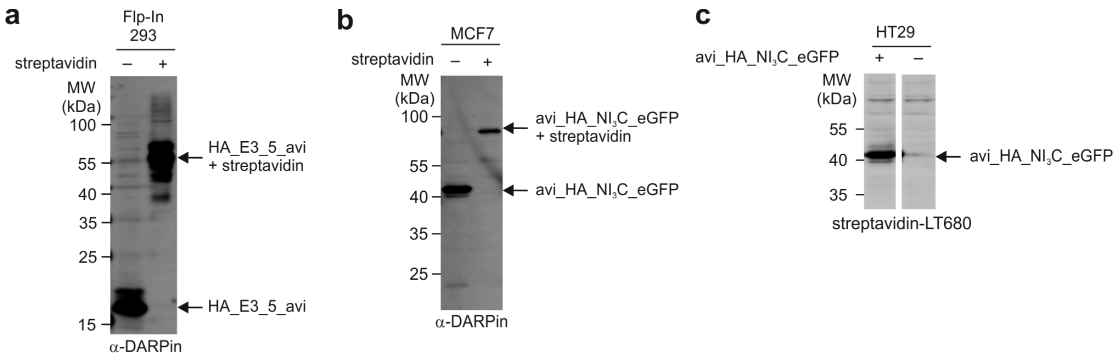

Supplementary Figure 1. Confirmation of the biotin ligase activity of stable cell lines overexpressing prokaryotic biotin ligase. (**a**) Flp-In 293 BirA cells were transiently transfected with HA_E3_5_avi and lysed 24 hours after transfection. The lysates were either pre-incubated with streptavidin (+) or left untreated (-) before being probed via western blotting using an anti-DARPin serum. (**b**) MCF7-BirA cells were transiently transfected with avi_HA_NI$_3$C_eGFP and further treated as described in (a). (**c**) HT29 BirA cells were transiently transfected with avi_HA_NI$_3$C_eGFP (+) or left untransfected (-). Since transfection levels (and thus protein levels) were very low for HT29 cells, the lysates were analyzed directly using fluorescently labeled streptavidin-LT680, which provides a more sensitive readout. The blot images have been cropped for conciseness and clarity. The full-size blots are presented in Supplementary Fig. 5.

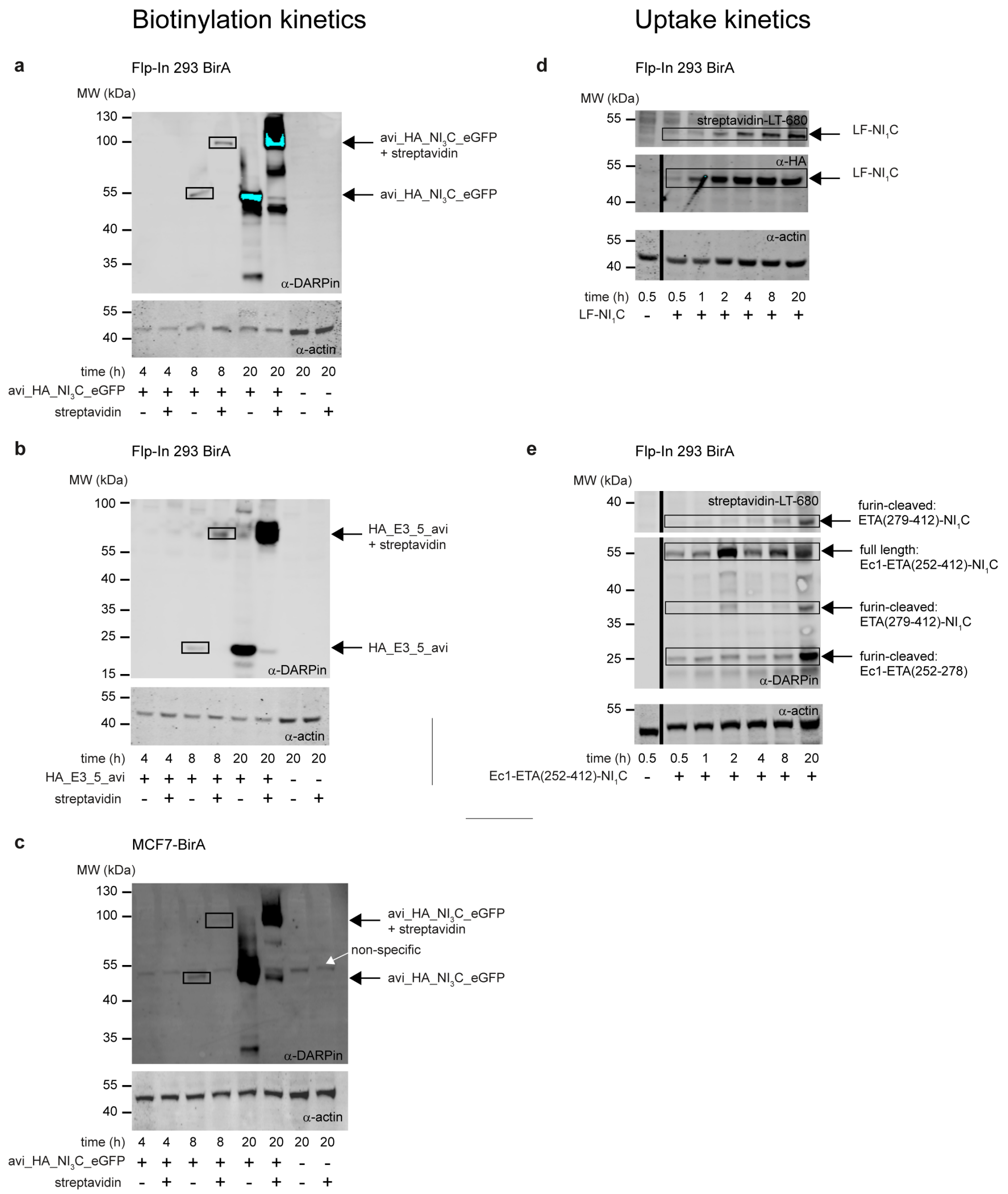

Supplementary Figure 2. Rate of cytosolic biotinylation and uptake to the cytosol. (a) Flp-In 293 BirA cells were transiently transfected with avi_HA_NI$_3$C_eGFP and lysed after the indicated timepoints. The lysates were either pre-incubated with streptavidin (+) or left untreated (-) before being probed via western blotting using anti-DARPin serum and an anti-actin antibody. Cyan indicates oversaturation of the signal. Note that the high intensity is required for the detection of the weak bands of interest at timepoint t = 8 h (boxed bands) and that the timepoint t = 20 h is of subordinate interest (b) Same experiment as in (a) for HA_E3_5_avi. (c) Same experiment as in (a) for MCF7-BirA cells. Please note that non-transfected cells were only treated with transfection reagent and were found to have a higher cell density at lysis, leading to more prominent actin bands for these samples. (d,e) Uptake kinetics for LF-NI$_1$C (d) and Ec1-ETA(252-412)-NI$_1$C (e) in Flp-In 293 BirA cells. Cells were incubated with 200 nM LF-NI$_1$C and 20 nM PA-Ac2 (transporter #11) or with 200 nM Ec1-ETA(252-412)-NI$_1$C (transporter #8) and lysed after the indicated timepoints. The lysates were then probed via western blotting using streptavidin LT-680, an anti-HA antibody, anti-DARPin serum or an anti-actin antibody. The blot images have been cropped for conciseness and clarity. The full-size blots are presented in Supplementary Fig. 5.

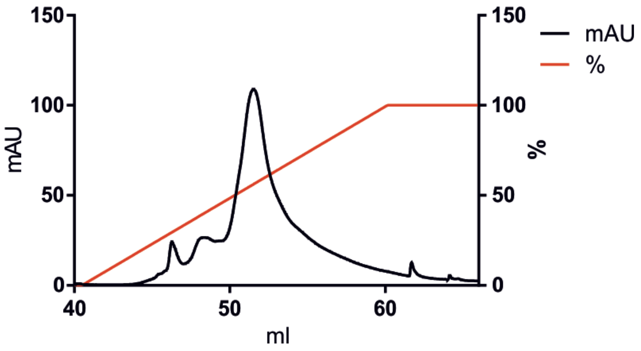

Supplementary Figure 3. Example chromatogram from the purification of scGFP by cation-exchange chromatography. The red line in the diagrams shows the percentage buffer B in a gradient going from PBS with 0.5 M NaCl (buffer A) to PBS with 2 M NaCl (buffer B). Typically, scGFP and fusions thereof eluted around 0.8-1.0 M NaCl.

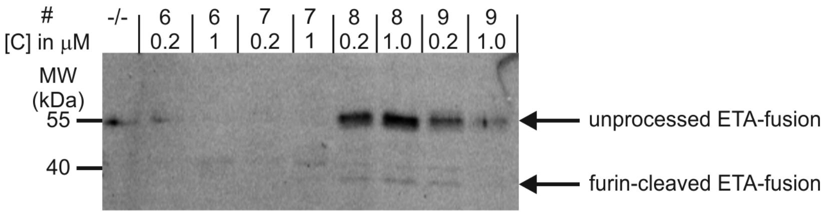

Supplementary Figure 4. Anti-DARPin western blot of uptake experiment in MCF7 cells. MCF7 cells were incubated with various cargo proteins for 20 h. Lysates were probed with anti-DARPin serum. Numbering of proteins according to Fig. 4. The blot image has been cropped for conciseness and clarity. The full-size blot is presented in Supplementary Fig. 5.

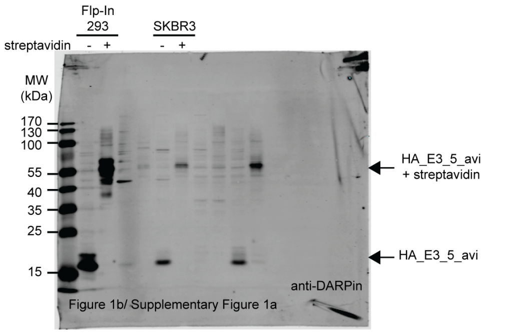
Flp-In 293
SKBR3
streptavidin - + - +
MW (kDa)
170
130
100
55
40
35
25
15
HA_E3_5_avi + streptavidin
HA_E3_5_avi
anti-DARPin
Figure 1b/ Supplementary Figure 1a

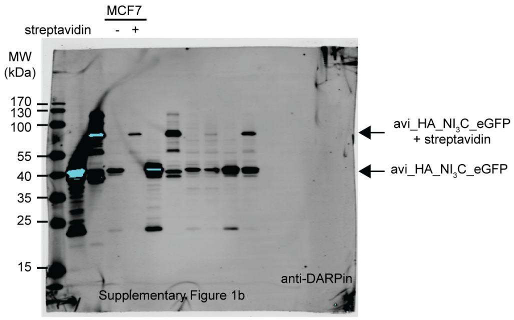
MCF7
streptavidin - +
MW (kDa)
170
130
100
55
40
35
25
15
avi_HA_NI$_3$C_eGFP + streptavidin
avi_HA_NI$_3$C_eGFP
anti-DARPin
Supplementary Figure 1b

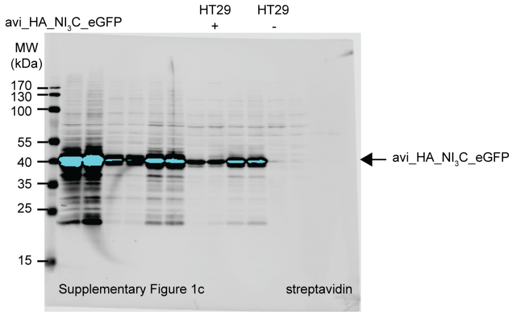
avi_HA_NI$_3$C_eGFP
HT29 +
HT29 -
MW (kDa)
170
130
100
55
40
35
25
15
avi_HA_NI$_3$C_eGFP
Supplementary Figure 1c
streptavidin

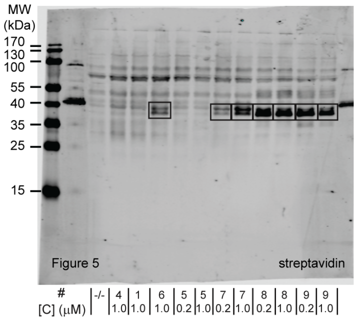
MW (kDa)
170
130
100
55
40
35
25
15
Figure 5
streptavidin
-/- 4 1 6 5 5 7 7 8 8 9 9
[C] (μM) 1.0 1.0 1.0 0.2 1.0 0.2 1.0 0.2 1.0 0.2 1.0

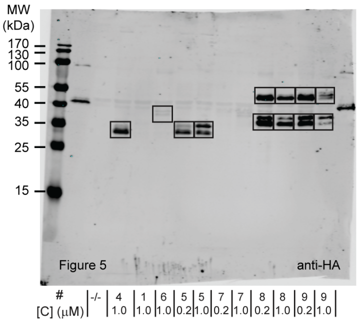
MW (kDa)
170
130
100
55
40
35
25
15
Figure 5
anti-HA
-/- 4 1 6 5 5 7 7 8 8 9 9
[C] (μM) 1.0 1.0 1.0 0.2 1.0 0.2 1.0 0.2 1.0 0.2 1.0

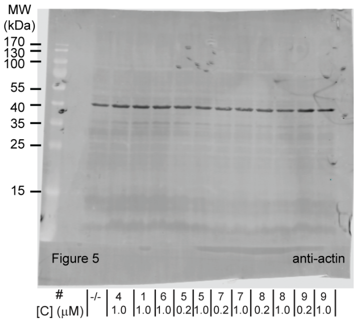
MW (kDa)
170
130
100
55
40
35
25
15
Figure 5
anti-actin
-/- 4 1 6 5 5 7 7 8 8 9 9
[C] (μM) 1.0 1.0 1.0 0.2 1.0 0.2 1.0 0.2 1.0 0.2 1.0

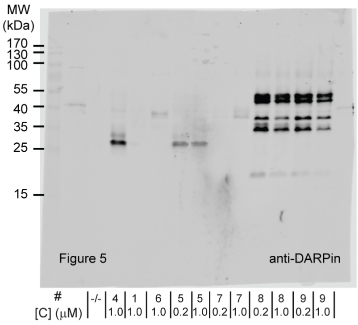
MW (kDa)
170
130
100
55
40
35
25
15
Figure 5
anti-DARPin
-/- 4 1 6 5 5 7 7 8 8 9 9
[C] (μM) 1.0 1.0 1.0 0.2 1.0 0.2 1.0 0.2 1.0 0.2 1.0

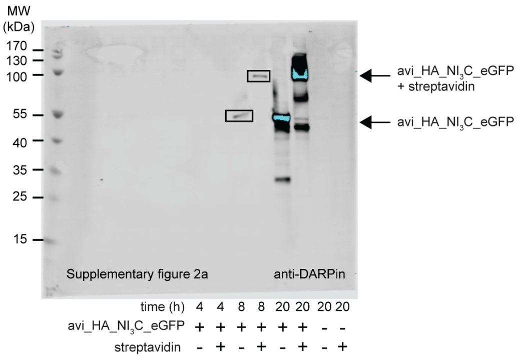
MW (kDa)
170
130
100
55
40
35
25
15
avi_HA_NI3C_eGFP + streptavidin
avi_HA_NI3C_eGFP
Supplementary figure 2a
anti-DARPin
time (h) 4 4 8 8 20 20 20 20
avi_HA_NI3C_eGFP + + + + + + - -
streptavidin - + - + - + - +

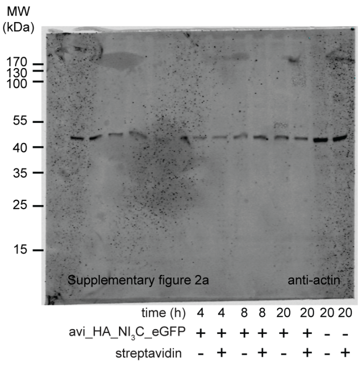
MW (kDa)
170
130
100
55
40
35
25
15
Supplementary figure 2a
anti-actin
time (h) 4 4 8 8 20 20 20 20
avi_HA_NI3C_eGFP + + + + + + - -
streptavidin - + - + - + - +

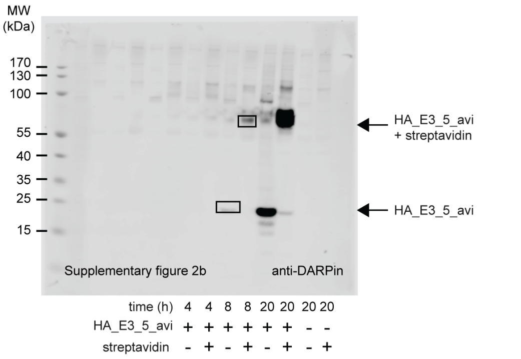
MW (kDa)
170
130
100
55
40
35
25
15
HA_E3_5_avi + streptavidin
HA_E3_5_avi
Supplementary figure 2b
anti-DARPin
time (h) 4 4 8 8 20 20 20 20
HA_E3_5_avi + + + + + + - -
streptavidin - + - + - + - +

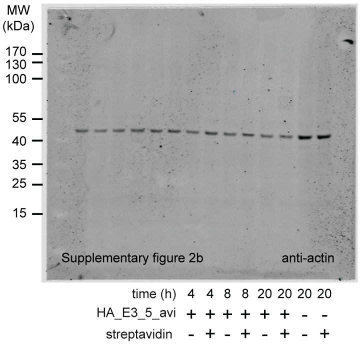
MW (kDa)
170
130
100
55
40
35
25
15
Supplementary figure 2b
anti-actin
time (h) 4 4 8 8 20 20 20 20
HA_E3_5_avi + + + + + + - -
streptavidin - + - + - + - +

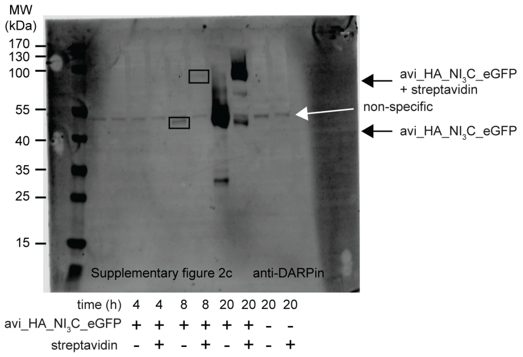
MW (kDa)
170
130
100
55
40
35
25
15
avi_HA_NI3C_eGFP + streptavidin
non-specific
avi_HA_NI3C_eGFP
Supplementary figure 2c
anti-DARPin
time (h) 4 4 8 8 20 20 20
avi_HA_NI3C_eGFP + + + + + + - -
streptavidin - + - + - + - +

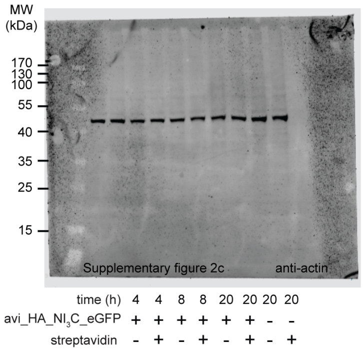
MW (kDa)
170
130
100
55
40
35
25
15
Supplementary figure 2c
anti-actin
time (h) 4 4 8 8 20 20 20 20
avi_HA_NI3C_eGFP + + + + + + - -
streptavidin - + - + - + - +

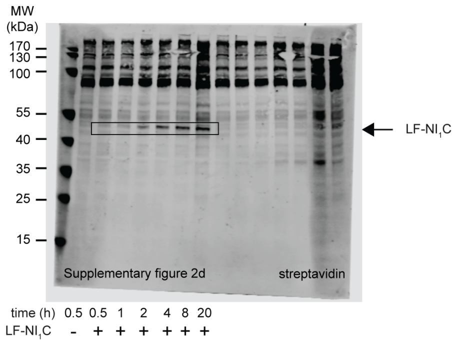
MW
(kDa)
170
130
100
55
40
35
25
15
LF-NI$_1$C
Supplementary figure 2d
streptavidin
time (h) 0.5 0.5 1 2 4 8 20
LF-NI$_1$C - + + + + + +

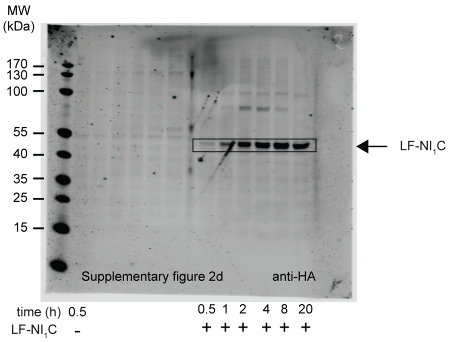
MW
(kDa)
170
130
100
55
40
35
25
15
LF-NI$_1$C
Supplementary figure 2d
anti-HA
time (h) 0.5 0.5 1 2 4 8 20
LF-NI$_1$C - + + + + + +

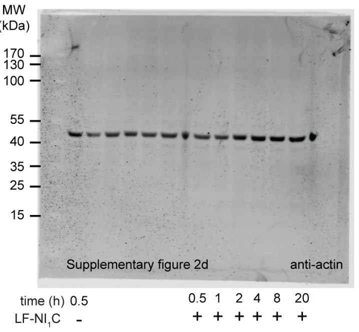
MW
(kDa)
170
130
100
55
40
35
25
15
Supplementary figure 2d
anti-actin
time (h) 0.5 0.5 1 2 4 8 20
LF-NI$_1$C - + + + + + +

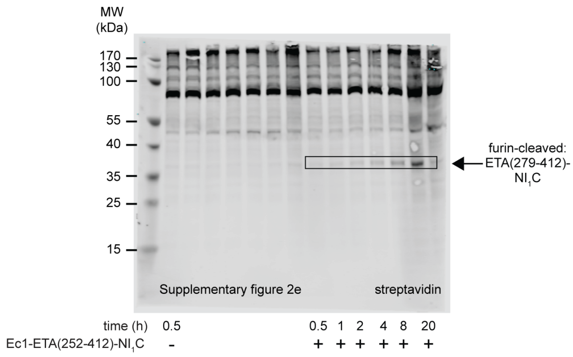
MW
(kDa)
170
130
100
55
40
35
25
15
furin-cleaved:
ETA(279-412)-
NI$_1$C
Supplementary figure 2e
streptavidin
time (h) 0.5 0.5 1 2 4 8 20
Ec1-ETA(252-412)-NI$_1$C - + + + + + +

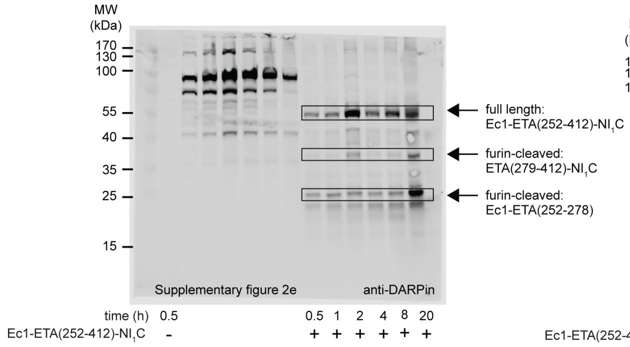
MW
(kDa)
170
130
100
55
40
35
25
15
full length:
Ec1-ETA(252-412)-NI$_1$C
furin-cleaved:
ETA(279-412)-NI$_1$C
furin-cleaved:
Ec1-ETA(252-278)
Supplementary figure 2e
anti-DARPin
time (h) 0.5 0.5 1 2 4 8 20
Ec1-ETA(252-412)-NI$_1$C - + + + + + +

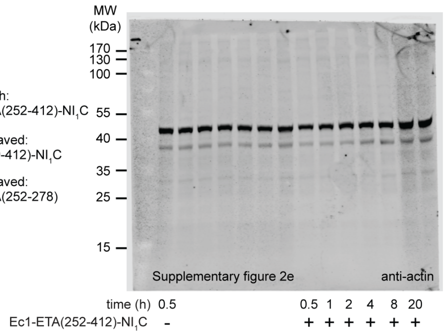
MW
(kDa)
170
130
100
55
40
35
25
15
Supplementary figure 2e
anti-actin
time (h) 0.5 0.5 1 2 4 8 20
Ec1-ETA(252-412)-NI$_1$C - + + + + + +

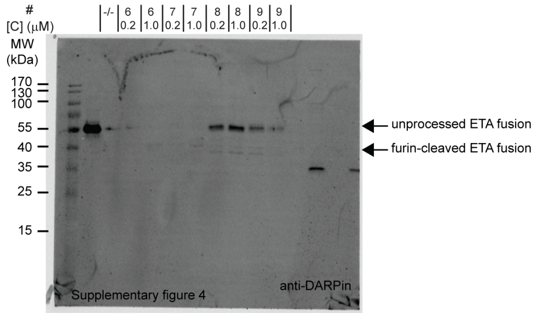
#
[C] (μM)
-/- 6 6 7 7 8 8 9 9
0.2 1.0 0.2 1.0 0.2 1.0 0.2 1.0
MW
(kDa)
170
130
100
55
40
35
25
15
unprocessed ETA fusion
furin-cleaved ETA fusion
Supplementary figure 4
anti-DARPin

Supplementary Figure 5. Full-size blot images of all blots present in the main part and the supplements. The figure numbers are indicated in the blots. The labeling is always the same for cropped and full-size blots.

Supplementary Table 1. Uptake levels of transport systems at additional external concentrations

Delivery system	External conc. (M)		MCF7<br>20 h, conc. (M)	SKBR3<br>20 h, conc. (M)	Flp-In 293<br>4 h, conc. (M)	Flp-In 293<br>20 h, conc. (M)
scGFP	1.0×10^{-6}	Cytosol	3.1×10^{-8}<br>[$1.2 \times 10^{-8} – 5.1 \times 10^{-8}$]	nd	1.3×10^{-6}<br>[$1.6 \times 10^{-8} – 3.8 \times 10^{-6}$]	2.7×10^{-7}<br>[$5.6 \times 10^{-8} – 4.8 \times 10^{-7}$]
	1.0×10^{-6}	Total cell	7.9×10^{-6}<br>[$5.4 \times 10^{-6} – 1.0 \times 10^{-5}$]	nd	2.6×10^{-5}<br>[$1.6 \times 10^{-5} – 3.6 \times 10^{-5}$]	6.6×10^{-5}<br>[$8.0 \times 10^{-6} – 1.2 \times 10^{-4}$]
Ec1-scGFP	1.0×10^{-6}	Cytosol	$0 – 4.9 \times 10^{-8}$	2.2×10^{-7}<br>[$6.9 \times 10^{-9} – 4.4 \times 10^{-7}$]	1.6×10^{-7}<br>[$1.2 \times 10^{-7} – 1.8 \times 10^{-7}$]	$0 – 9.4 \times 10^{-8}$
	1.0×10^{-6}	Total cell	3.6×10^{-6}<br>[$1.6 \times 10^{-6} – 5.6 \times 10^{-6}$]	2.8×10^{-6}<br>[$9.2 \times 10^{-7} – 4.7 \times 10^{-6}$]	1.2×10^{-5}<br>[$1.0 \times 10^{-5} – 1.3 \times 10^{-5}$]	1.3×10^{-5}<br>[$3.3 \times 10^{-6} – 3.0 \times 10^{-5}$]
Tat-NI_1C	2.0×10^{-7}	Cytosol	nd	0	7.8×10^{-9}<br>[$3.9 \times 10^{-9} – 1.2 \times 10^{-8}$]	0
	2.0×10^{-7}	Total cell	nd	0	1.4×10^{-8}<br>[$5.9 \times 10^{-9} – 2.1 \times 10^{-8}$]	0
Penetratin-NI_1C	2.0×10^{-7}	Cytosol	nd	0	2.5×10^{-8}<br>[$2.5 \times 10^{-9} – 4.8 \times 10^{-8}$]	0
	2.0×10^{-7}	Total cell	nd	0	4.5×10^{-8}<br>[$1.4 \times 10^{-8} – 7.8 \times 10^{-8}$]	3.4×10^{-8}<br>[$1.6 \times 10^{-8} – 5.3 \times 10^{-8}$]
Ac2-DT-NI_1C	2.0×10^{-7}	Cytosol	0	0	0	0
	2.0×10^{-7}	Total cell	0	0	0	0

Values in brackets below the average value indicate ranges observed; nd = not determined

Supplementary Table 2. Detection of cytosolically delivered cargo (biotin) as a fraction of the amount of cargo detected via the HA tag, which reflects total cell internalization

Delivery system	Conc. (M)	MCF7<br>20 h	SKBR3<br>20 h	HT29<br>20 h	Flp-In 293<br>4 h	Flp-In 293<br>20 h
Penetratin-NI_1C	5.0×10^{-6}					2 – 5
ETA- NI_1C	2.0×10^{-7}			100*		
ETA- NI_1C	1.0×10^{-6}			100*		100*
Tat-ETA- NI_1C	2.0×10^{-7}				61 – 100*	100*
Tat-ETA- NI_1C	1.0×10^{-6}			100*	100*	100*
Ec1-ETA- NI_1C	2.0×10^{-7}			100*	11 – 100*	100*
Ec1-ETA- NI_1C	1.0×10^{-6}			100*	26 – 100*	100*
Tat- Ec1-ETA- NI_1C	2.0×10^{-7}			100*	14 – 100*	100*
Tat- Ec1-ETA- NI_1C	1.0×10^{-6}			100*	36 – 100*	100*
Ac2-DT- NI_1C	1.0×10^{-6}				3 – 24	
Ac2-DT- NI_1C	5.0×10^{-6}				1.5 – 5	17 – 100*
LF- NI_1C/NI_2C-dest.5 + PA-Ac2	2.0×10^{-7} + 2.0×10^{-8}			11 – 100*	19 – 19	24 – 28
LF- NI_1C/ NI_2C-dest.5 + PA-sAntrx-Ac2	2.0×10^{-7} + 2.0×10^{-8}			52 – 100*	11 – 19	17 – 27
scGFP	2.0×10^{-7}	0.2 – 0.4	0.3 – 0.7	0.5 – 1.1	0.0 – 6	0.7 – 8
scGFP	1.0×10^{-6}	0.1 – 0.6			0.1 – 10	0.2 – 0.7
scGFP- NI_1C	2.0×10^{-7}		1.0 – 1.9	0.3 – 3		
Ec1-scGFP	2.0×10^{-7}		1.9 – 3	0.5 – 1.6		
Ec1-scGFP	1.0×10^{-6}		0.7 – 9			

* 100 indicates that the calculated amount of protein using the biotin signal was higher than the calculated amount of protein using the HA signal

Supplementary Table 3. Estimation of total cellular internalization in MCF7 cells using the anti-DARPin serum

Delivery system	External conc. (M)	Total cell conc. (M) based on DARPin signal; [Range]
Ec1-ETA-NI_1C	2.0×10^{-7}	7.8×10^{-8} [3.0×10^{-8} – 2.5×10^{-7}]
Ec1-ETA-NI_1C	1.0×10^{-6}	1.1×10^{-7} [4.8×10^{-8} – 3.4×10^{-7}]
Tat-Ec1-ETA-NI_1C	2.0×10^{-7}	5.9×10^{-8} M [1.7×10^{-8} – 2.0×10^{-7}]
Tat-Ec1-ETA-NI_1C	1.0×10^{-6}	3.0×10^{-8} [1.3×10^{-8} – 9.0×10^{-8}]
NI_1C-DT-Ac2	2.0×10^{-7}	1.1×10^{-8} [4.7×10^{-9} – 1.5×10^{-8}]
NI_1C-DT-Ac2	1.0×10^{-6}	2.0×10^{-8} [5.9×10^{-9} – 2.9×10^{-8}]
NI_1C-DT-Ac2	5.0×10^{-6}	4.0×10^{-8} [7.1×10^{-9} – 6.1×10^{-8}]

Supplementary Table 4. Ratio of total cell concentration calculations using the anti-HA antibody or the anti-DARPin serum

Flp-In 293	External conc. (M)	Ratio HA/DARPin (4 h) [range]	Ratio HA/DARPin (20 h) [range]
Ec1-ETA-NI_1C	2.0×10^{-7}	0.23 [0.17-0.29]	0.88 [0.35-1.35]
Ec1-ETA-NI_1C	1.0×10^{-6}	0.18 [0.10-0.27]	0.60 [0.44-0.69]
Tat-Ec1-ETA-NI_1C	2.0×10^{-7}	0.31 [0.12-0.55]	0.79 [0.46-1.11]
Tat-Ec1-ETA-NI_1C	1.0×10^{-6}	0.33 [0.05-0.36]	0.77 [0.43-1.10]

References

1. Wetzel, S.K., Settanni, G., Kenig, M., Binz, H.K. & Plückthun, A. Folding and unfolding mechanism of highly stable full-consensus ankyrin repeat proteins. *J. Mol. Biol.* **376**, 241-257 (2008).
2. Verdurmen, W.P., Luginbühl, M., Honegger, A. & Plückthun, A. Efficient cell-specific uptake of binding proteins into the cytoplasm through engineered modular transport systems. *J Control Release* **200**, 13-22 (2015).
3. McNaughton, B.R., Cronican, J.J., Thompson, D.B. & Liu, D.R. Mammalian cell penetration, siRNA transfection, and DNA transfection by supercharged proteins. *Proc. Natl. Acad. Sci. U. S. A.* **106**, 6111-6116 (2009).
